# Iron Metabolism in Aminolevulinic Acid-Photodynamic Therapy with Iron Chelators from the Thiosemicarbazone Group

**DOI:** 10.3390/ijms251910468

**Published:** 2024-09-28

**Authors:** Robert Gawecki, Patrycja Rawicka, Marta Rogalska, Maciej Serda, Anna Mrozek-Wilczkiewicz

**Affiliations:** 1 Institute of Physics, University of Silesia in Katowice, 75 Pułku Piechoty 1A, 41-500 Chorzow, Poland; robert.gawecki@us.edu.pl (R.G.); patrycja.rawicka@us.edu.pl (P.R.); 2 SPIN-Lab Centre for Microscopic Research of Matter, University of Silesia in Katowice, 75 Pułku Piechoty 1A, 41-500 Chorzow, Poland; 3 Institute of Chemistry, University of Silesia in Katowice, Szkolna 9, 40-006 Katowice, Poland; rejmund.m@gmail.com (M.R.); maciej.serda@us.edu.pl (M.S.); 4 Department of Systems Biology and Engineering, Silesian University of Technology, Akademicka 16, 44-100 Gliwice, Poland

**Keywords:** iron metabolism, protoporphyrin IX, 5-aminolevulinic acid, photodynamic therapy, thiosemicarbazone, iron chelators

## Abstract

Iron plays a crucial role in various metabolic processes. However, the impact of 5-aminolevulinic acid (ALA) in combination with iron chelators on iron metabolism and the efficacy of ALA-photodynamic therapy (PDT) remain inadequately understood. This study aimed to examine the effect of thiosemicarbazone derivatives during ALA treatment on specific genes related to iron metabolism, with a particular emphasis on mitochondrial iron metabolism genes. In our study, we observed differences depending on the cell line studied. For the HCT116 and MCF-7 cell lines, in most cases, the decrease in the expression of selected targets correlated with the increase in protoporphyrin IX (PPIX) concentration and the observed photodynamic effect, aligning with existing literature data. The Hs683 cell line showed a different gene expression pattern, previously not described in the literature. In this study, we collected an extensive analysis of the gene variation occurring after the application of novel thiosemicarbazone derivatives and presented versatile and effective compounds with great potential for use in ALA-PDT.

## 1. Introduction

**Figure 1:**
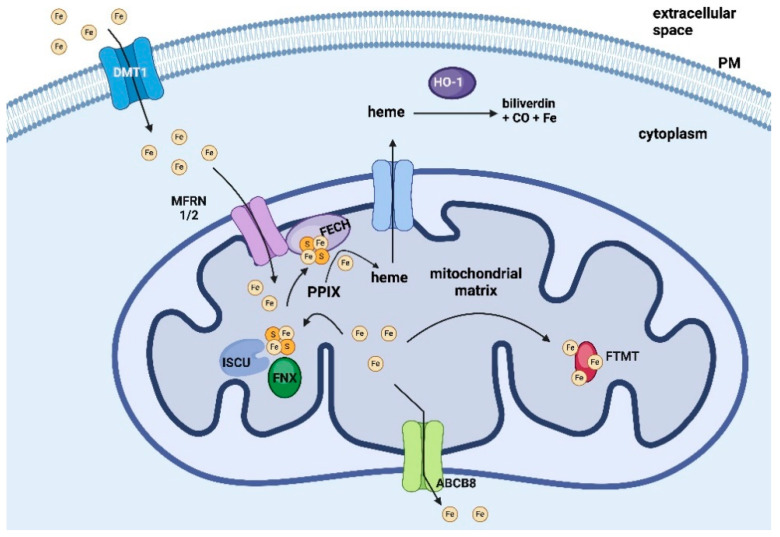
Iron metabolism in a cell considering the molecular targets studied in this paper (created in BioRender). PPIX—protoporphyrin IX, FECH—ferrochelatase, HO-1—heme oxygenase, FTMT—ferritin, FXN—frataxin, ICSU—iron-sulfur cluster assembly enzyme, MFRN1/2—mitoferrin 1/2, ABCB8—ATP-binding cassette subfamily B member 8.

Photodynamic therapy (PDT), a method characterized by its low invasiveness towards healthy cells, is currently an attractive treatment strategy for various diseases. Despite its great potential against cancer cells, it is mainly used for dermatological treatment and cosmetic procedures. Due to the vast variability within a single cancer type, as well as the individual characteristics of each patient, current treatment methods need to be modified. Therefore, developing non-destructive methods of cancer treatment for the patient is particularly important. In this paper, we present a modification of PDT using 5-aminolevulinic acid (5-ALA) supported by iron chelators from the thiosemicarbazone (TSC) group. Although this strategy is well-known [1,2], there is a paucity of data on the changes in iron metabolism that occur during this therapy. The therapy itself consists of administering 5-ALA, a precursor of the actual drug—the photosensitizer protoporphyrin IX (PPIX), which, when excited with harmless radiation in the red wavelength range, generates reactive oxygen species (ROS) that initiate radical reactions leading to tumor destruction [3]. Using chelators, it is possible to increase the concentration of PPIX by blocking its conversion to heme [4,5]. Despite the use of different classes of chelators such as deferoxamine (DFO) [6], ethylenediaminetetraacetic acid (EDTA) [7], AP2-18 [8], and Cp94 [9], this therapy has not received much attention. In this work, we present a new group of chelators that effectively increase drug concentration, thereby affecting several cellular pathways. However, as previously mentioned, little is known about the changes that occur in iron metabolism, so in this work, we have performed an extensive analysis of gene variation of iron-management-related targets. In the recent study, we selected molecular targets (Figure 1) related to iron management.

Ferrochelatase (FECH) is considered one of the key genes affecting the efficiency of PPIX accumulation and the efficacy of ALA-PDT. This enzyme catalyzes the incorporation of an iron ion into PPIX, leading to the formation of heme. Numerous studies have demonstrated that reduced expression of this gene positively impacts PPIX accumulation and enhances the efficacy of ALA-PDT [10,11]. Another gene frequently studied recently is heme oxygenase (HO-1), an enzyme that degrades heme into biliverdin, carbon monoxide, and iron ions. Downregulation of HO-1 expression has been shown to enhance ALA-PDT efficiency in several cell lines, likely due to its role in protection against ROS [12]. Mitochondrial ferritin (FTMT) is a small protein with structural similarities to cytosolic heavy ferritin (HFt), found within the mitochondrial matrix. Functionally, FTMT serves as a storage site for mitochondrial iron and exhibits ferroxidase activity [13]. Another equally important molecule associated with iron metabolism is frataxin (FXN), which plays a crucial role in the biosynthesis of [2Fe-2S]-type iron centers. FXN is presumed to engage in physical interactions with the iron-sulfur cluster assembly enzyme (ICSU), serving as the primary sulfur donor in the biosynthesis of iron-sulfur clusters. However, the precise role of this protein remains largely unexplored [14,15]. Mitoferrin 1 and 2 (MFRN1 and MFRN2, now SLC25A37 and SLC25A28, respectively) serve as iron transporters, facilitating the transport of iron from the cytoplasm to the mitochondria. These transporters are located in the inner mitochondrial membrane. While both forms of mitoferrin are found in various tissues, MFRN1 exhibits significantly higher expression levels in erythroid tissue cells [16]. Furthermore, it has been demonstrated that MFRN1 forms a complex with ABCB10 and FECH. In this interaction, MFRN1 is directly implicated in transporting iron to the active center of FECH [17]. Another important iron transporter is divalent metal transporter 1 (DMT1, now SLC11A2), which facilitates the cellular uptake of iron ions [18]. While DMT1 is predominantly localized in the cell membrane, reports suggest its potential involvement in the transport of iron ions within the mitochondria as well [19,20]. To comprehensively trace the fate of the iron ion in the cell, we also included the target responsible for its efflux in the analysis. Namely, we focused on the mitochondrial potassium channel ATP-binding subunit (ABCB8) gene, which is involved in the mitochondrial-to-cytosolic iron export process and the maturation of cytosolic iron-sulfur cluster-containing enzymes [21].

In our previous work, we have described the TSC-induced changes in the cellular heme biosynthesis and degradation [22]. Interestingly, we observed significant differences depending on the cell line studied, with changes occurring in a manner not previously described in the literature. While the crucial roles of FECH and HO-1 are well-known, their upregulation has not been previously observed. In our subsequent work, we have gone a step further; namely, extending the analysis to other genes related to the transport of the heme substrates and heme itself [23]. These results proved that, despite the upregulation of the ABCG2 transporter involved in the PPIX efflux, our TSCs increased PPIX levels sufficiently to trigger a therapeutic effect. This demonstrates that these compounds have great potential as supportive therapy for ALA-PDT. We also observed interesting changes in the iron transporter MFRN1 behavior; therefore, we have extended the study of its analogs in this paper.

Thiosemicarbazones itself are very interesting compounds known for their unique physicochemical properties, which arise from the presence of the TSC functional group. These properties are influenced by the ability of TSC to form stable complexes with metal ions due to their sulfur and nitrogen donor atoms. They exhibit varied solubility in polar solvents, stability under different pH conditions, and distinct melting points. Additionally, their structural flexibility allows for modifications, which enhances their biological and pharmacological activities, making them of significant interest in medicinal chemistry and material science.

## 2. Results and Discussion

### 2.1. Synthesis

**Figure 2:**
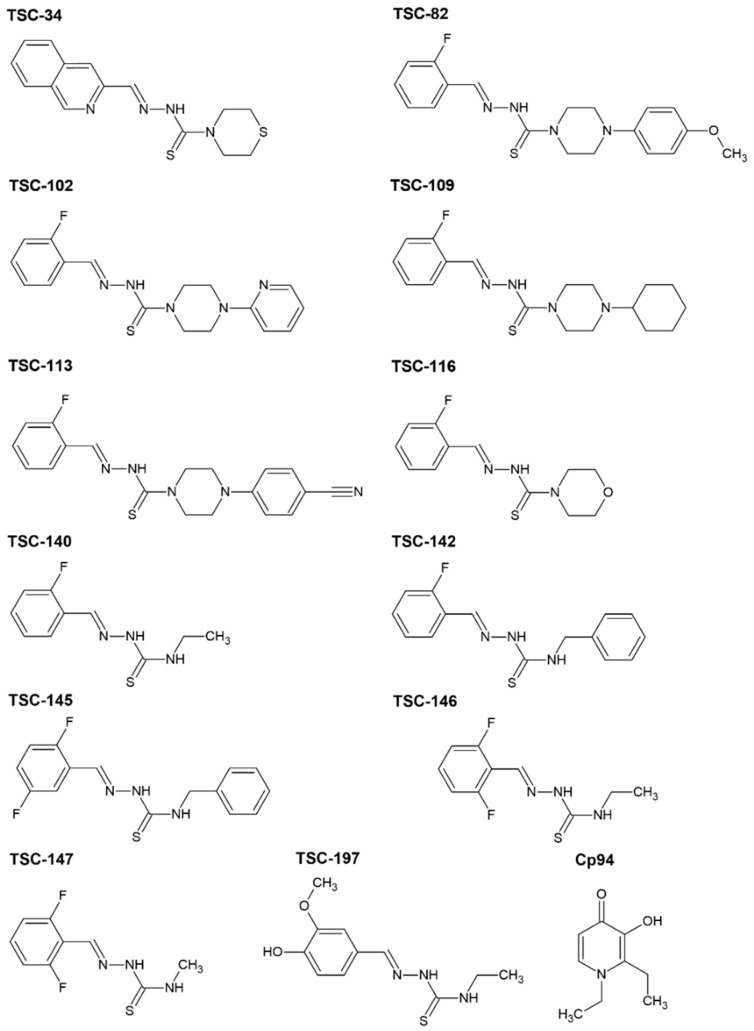
The structures of the TSCs and Cp94 examined in this study.

Aromatic TSCs were synthesized according to the methodology previously published by our group and depicted below (Figure 2) [24]. The aliphatic TSC precursors were obtained from commercial resources. However, aromatic TSCs were synthesized in our laboratories. Derivative TSC-34 was described and further investigated by us in [22,23,25]. TSC-146 and TSC-147 were commercially available. TSC-197 was published in [26,27]. The other compounds are newly synthesized.

### 2.2. The Antiproliferative Activity of TSC

As was demonstrated in our previous research, chelators within the TSC group exhibit significant potential for enhancing the efficacy of ALA-PDT [22]. In this study, we selected a set of compounds (Figure 1) from this group to explore their influence on ALA-PDT efficiency and their effects on iron metabolism within malignant cell lines. TSCs are recognized for their diverse range of biological activities. In our studies, the goal was to select compounds that do not inherently possess pro-apoptotic or cell-proliferation-inhibiting effects. This decision stems from the understanding that cytotoxic compounds within the TSC group do not induce an increase in PPIX accumulation [28], which is a fundamental prerequisite for the action of ALA-PDT. In this study, we examined the effects of TSCs and Cp94 on three cell lines of different origins. As illustrated in Table 1, none of these compounds exhibited antiproliferative activity against the tested cancer cell lines. Furthermore, they showed no activity against normal human fibroblasts, which is crucial for the selectivity of this therapy. The utilization of these chelators for PDT is justified by their potential to enhance the photodynamic effect without exhibiting inherent dark toxicity. In the majority of studies involving iron chelators, the chelators are administered at doses that do not adversely affect cell viability [29,30,31]. In our approach, we chose chelators from the TSC group at a concentration of 25 µM and Cp94 at the same concentration for comparison.

### 2.3. An Accumulation of PPIX

**Figure 3:**
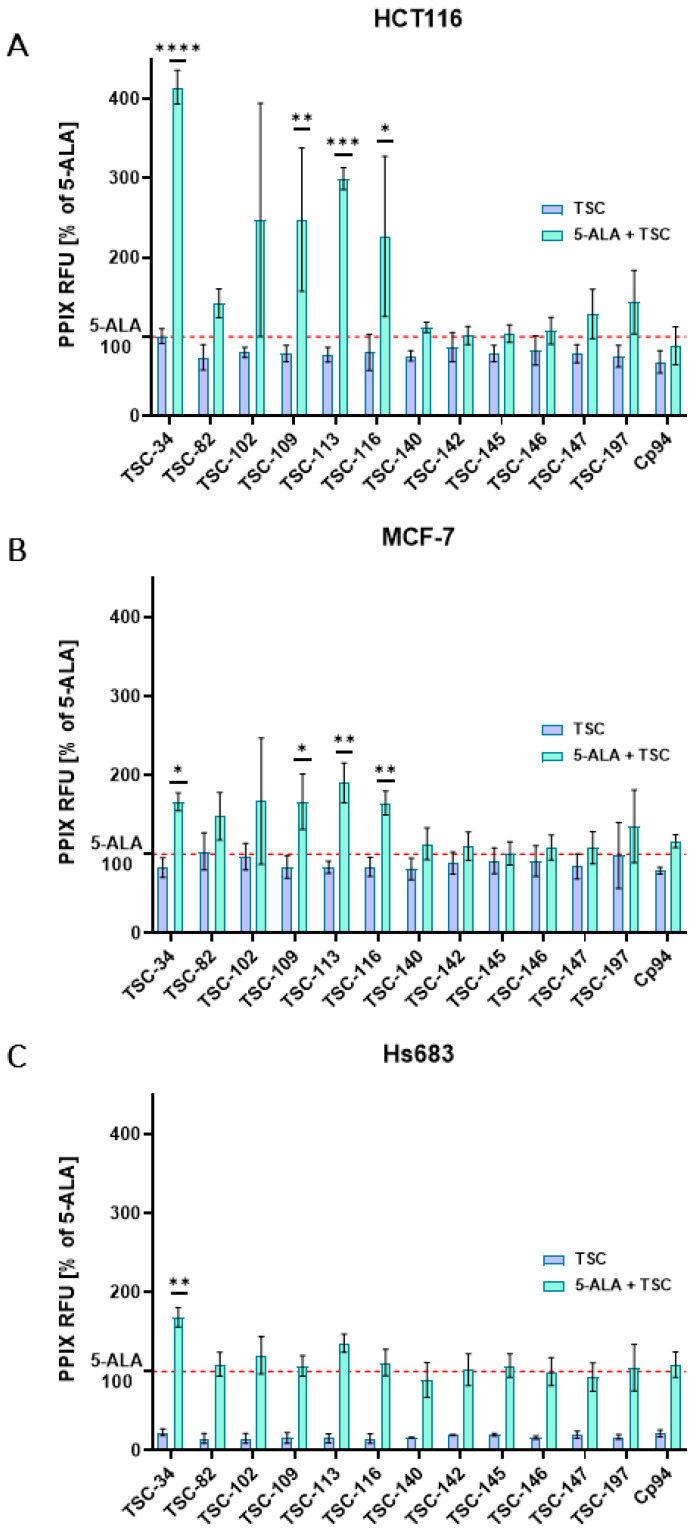
Accumulation of PPIX after treatment with 5-ALA, TSCs, Cp94, and their combinations on (**A**): HCT116, (**B**): MCF-7, and (**C**): Hs683 cell lines. The red dotted line on the graph depicts the fluorescence intensity of PPIX following treatment with 5-ALA alone. The data are presented as the means ± standard deviation from three independent experiments and analyzed using one-way ANOVA with Tukey’s post hoc test, indicating significance levels as follows: * *p* < 0.05, ** *p* < 0.01, *** *p* < 0.001, **** *p* < 0.0001.

To select promising TSC derivatives for improving ALA-PDT efficiency, we examined the fluorescence intensity of PPIX. The results clearly show that only some derivatives significantly increase the fluorescence intensity of PPIX after 5-ALA treatment in combination with TSC. In the HCT116 and MCF-7 cell lines, significantly elevated PPIX levels were observed after treatment with several derivatives (Figure 3A,B). All the data presented in Figure 3 represent statistically significant fold-changes relative to 5-ALA. For the HCT116 line, 4.14, 2.47, 3.0, and 2.26-fold changes were observed with the combination of 5-ALA and TSC-34, TSC-109, TSC-113, and TSC-116, respectively, compared to 5-ALA treatment alone. In the MCF-7 line, the fold increases were lower: 1.66, 1.64, and 1.9-fold increases for TSC-34, TSC-109, TSC-116, and TSC-113, respectively, in combination with 5-ALA. No significant changes were observed after treatment with Cp94 and 5-ALA in both cell lines. For the Hs638 line, a significant increase in PPIX fluorescence intensity was observed only with the combination of TSC-34 and 5-ALA. Similar to the other cell lines, no increase in PPIX intensity was observed for cells treated with Cp94 and 5-ALA (Figure 3C). The use of chelators to increase PPIX accumulation is not a new concept. Early chelators included compounds such as EDTA and DFO, which significantly raised PPIX levels in various cells. However, EDTA binds a variety of biologically important ions and can also raise PPIX levels in healthy tissues. On the other hand, DFO is an iron-selective chelator, but its large, lipophilic structure hinders cellular uptake [4]. Cp94, which lacks these drawbacks, has been shown to significantly raise PPIX levels in various cell lines. However, in our experiments, increased PPIX levels were not observed in any cell line treated with Cp94. This discrepancy can be explained by the concentration of Cp94 used, which was 25 µM. Many studies that reported significant PPIX increases used concentrations above 100 µM [8,31]. Considering this, the use of TSC derivatives is much more effective than Cp94.

### 2.4. An Ability to Chelate Iron Ions

**Figure 4:**
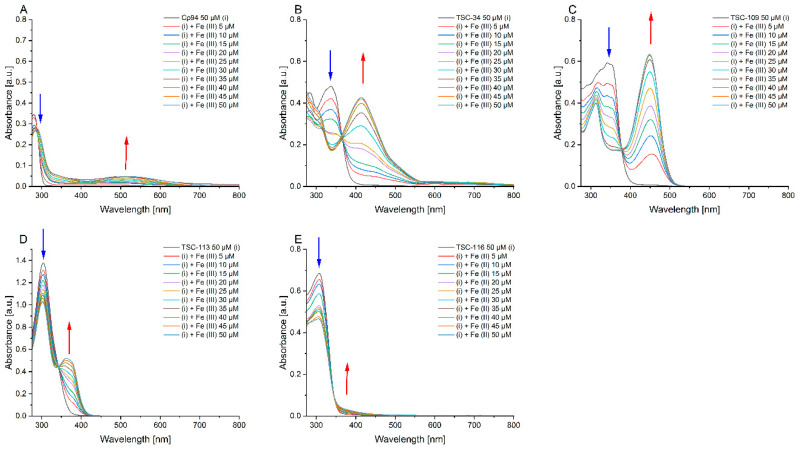
Absorption titration spectra of selected compounds (50 μM) with iron (III) ions in water: (**A**)—Cp94; (**B**)—TSC-34; (**C**)—TSC-109; (**D**)—TSC-113; (**E**)—TSC-116. Blue arrows indicate a decrease in band intensity for the test compound, while red arrows highlight an increase in band intensity for the complex. Measurements were performed at room temperature 4 h after the samples had been prepared.

Although TSCs are known for their ability to chelate iron ions, our compounds are novel. Therefore, to test their iron-chelating capabilities, we examined all compounds that caused an increase in PPIX fluorescence intensity. Spectroscopic measurements were conducted in an aqueous environment to closely mimic cellular conditions. The recorded absorption spectra, shown in Figure 4, illustrate the interaction with iron (III) ions, maintaining a constant compound concentration while varying the ion concentration. An isosbestic point was observed for all studied compounds, indicating the existence of at least two forms: free and bound with metal ions. The spectra revealed a decrease in the characteristic absorption band of each compound as the concentration of iron ions increased. Additionally, a new band emerged upon the addition of iron ions, with its absorbance increasing proportionally to the metal ion concentration. This new band likely corresponds to the formation of a complex between the tested compound and the metal ions. The specific positions of absorption maxima for each band and the isosbestic point are detailed in Table 2.

Compounds in the TSC group are widely studied, particularly for their anticancer activity. One of the most well-researched compounds is Dp44mT due to its ability to chelate iron ions. This compound has been examined in various environments. In our study, we observe the appearance of an isosbestic point, the emergence of a new band from the complex of the compound with iron, and the disappearance of a band associated with the free compound. These observations confirm that the TSCs studied also chelate iron ions in aqueous environments [32,33,34].

### 2.5. The Phototoxic Effect

**Figure 5:**
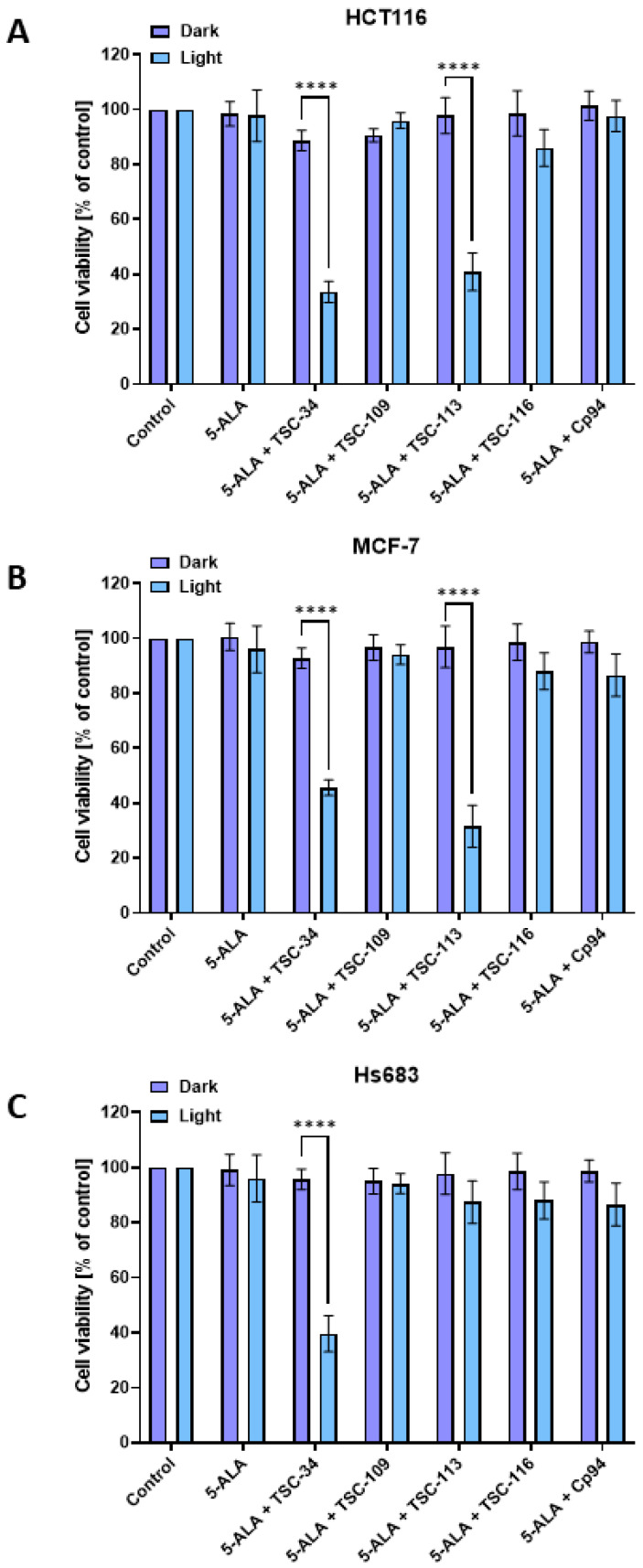
Phototoxic effect after treatment with 5-ALA, TSCs, Cp94, and their combinations on (**A**): HCT116, (**B**): MCF-7, and (**C**): Hs683 cell lines. The data are presented as means ± standard deviation from three independent experiments and analyzed using one-way ANOVA with Tukey’s post hoc test, indicating significance levels as follows: **** *p* < 0.0001.

After the accumulation experiments and confirming the selected compounds’ chelating ability, the tested compounds’ phototoxic effect in combination with 5-ALA was assessed. No significant decrease in cell viability was observed for any compound in any cell line without light (dark toxicity). However, after cell irradiation with red light, significant decreases in cell viability were observed for TSC-34 in combination with 5-ALA on the HCT116 and MCF-7 cell lines, with surviving fractions of 36.6% and 45.6%, respectively. Treatment with TSC-113 also significantly decreased the cell viability of the HCT116 and MCF-7 cell lines, with surviving fractions of 40.8% and 31.5%, respectively. In contrast, for the Hs683 cell line, a decrease in viability was observed only after treatment with TSC-34 in combination with 5-ALA, resulting in a surviving fraction of 39.5% (Figure 5C). No decrease in cell viability was observed in any cell line tested after treatment with Cp94 in combination with 5-ALA.

These results demonstrate the efficacy of our compounds against cancer cells in ALA-PDT therapy. Despite using a relatively low concentration of TSCs (25 µM), a therapeutic effect was still achieved. The tested TSCs effectively inhibited the growth of irradiated cells, in contrast to the Cp94 reference.

### 2.6. Iron Metabolism

**Figure 6:**
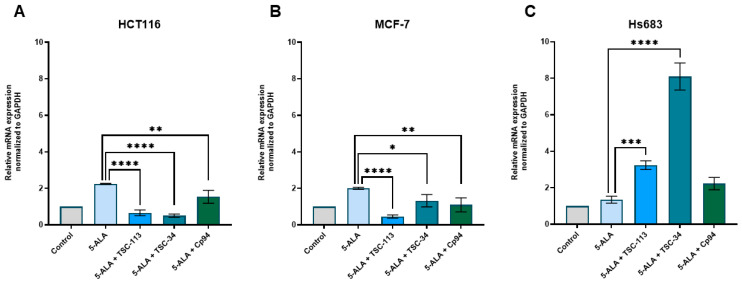
Expression of FECH in the (**A**): HCT116, (**B**): MCF-7, and (**C**): Hs683 cell lines. The data are presented as means ± standard deviation from three independent experiments and analyzed using one-way ANOVA with Tukey’s post hoc test, indicating significance levels as follows: * *p* < 0.05, ** *p* < 0.01, *** *p* < 0.001, **** *p* < 0.0001.

An experiment in which FECH expression was measured demonstrated that for the HCT116 and MCF-7 cell lines, a decrease in FECH expression was observed after treatment with TSC-34 and TSC-113 (Figure 6A,B). This aligns with literature reports and may explain the significant photocytotoxic effect on these cell lines. In contrast, for the Hs683 cell line, we observed an increase in FECH expression after treatment with TSC-34 and TSC-113 (Figure 6C). Despite the expected absence of a phototoxic effect on this cell line, a strong cytotoxic effect was observed after treatment with TSC-34. Additionally, in the HCT116 and MCF-7 cell lines treated with Cp94, a decrease in FECH expression was also observed, yet no phototoxic effect was noted. As demonstrated, elevated expression of FECH correlates with reduced PPIX accumulation. Conversely, diminished FECH expression leads to increased PPIX accumulation induced by 5-ALA treatment in various cancer cell lines [10]. In another study where FECH expression was repressed, untreated cells exhibited heightened PPIX levels, while no significant changes in PPIX accumulation were observed after 5-ALA treatment [35]. This observation suggests that FECH expression is not the sole factor in effective ALA-PDT, indicating that other factors must influence ALA-PDT efficacy.

**Figure 7:**
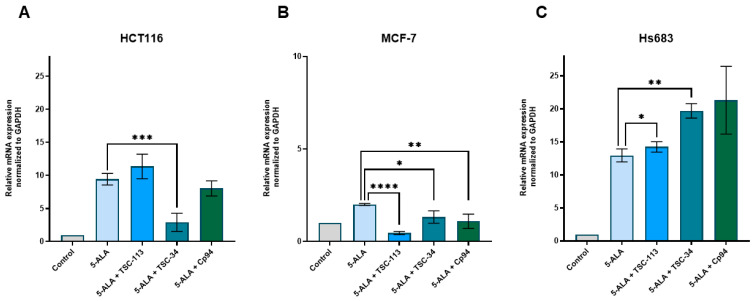
Expression of HO-1 in the (**A**): HCT116, (**B**): MCF-7, and (**C**): Hs683 cell lines. The data are presented as means ± standard deviation from three independent experiments and analyzed using one-way ANOVA with Tukey’s post hoc test, indicating significance levels as follows: * *p* < 0.05, ** *p* < 0.01, *** *p* < 0.001, **** *p* < 0.0001.

The MCF-7 cell line exhibited a decrease in HO-1 expression after treatment with all TSC derivatives, as well as Cp94 (Figure 7B). For the HCT116 cell line, we observed a decrease in HO-1 expression only with TSC-34 treatment (Figure 7A). In the case of the Hs683 cell line (Figure 7C), our observations contrasted with literature findings. Increased levels of HO-1 are typically associated with low ALA-PDT efficiency [12], which may explain the lack of a phototoxic effect after TSC-113 treatment. However, this does not account for the strong phototoxic effect observed with TSC-34 treatment in this cell line. Combining the results obtained for FECH and HO-1, we can conclude that the reduced expression of FECH, leading to increased PPIX accumulation along with decreased protection against ROS due to reduced HO-1 expression, explains the strong phototoxic effects observed after TSC-34 treatment in the HCT116 and MCF-7 cell lines. However, the opposite results for the Hs683 line do not explain the effective response to TSC-34 treatment, although they may account for the lack of response after TSC-113 treatment.

**Figure 8:**
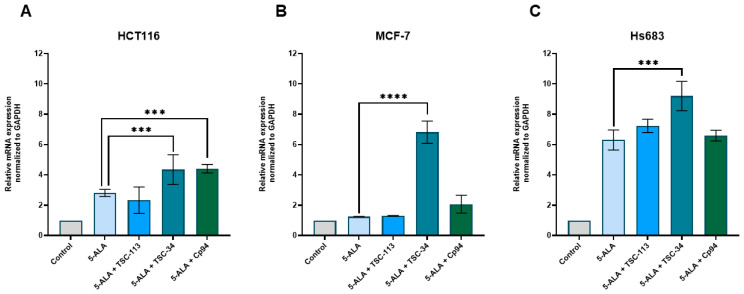
Expression of FTMT in the (**A**): HCT116, (**B**): MCF-7, and (**C**): Hs683 cell lines. The data are presented as means ± standard deviation from three independent experiments and analyzed using one-way ANOVA with Tukey’s post hoc test, indicating significance levels as follows: *** *p* < 0.001, **** *p* < 0.0001.

A significant upregulation of FTMT expression in cells treated with the combination of TSC-34 and 5-ALA across all cell lines was observed (Figure 8). Notably, for the HCT116 cell line, increased expression of FTMT was also noted after treatment with Cp94 and 5-ALA. As demonstrated, disruption of iron availability using deferiprone causes an increase in FTMT expression [36]. Elevated levels of this protein induce mitochondrial damage and depolarization of the mitochondrial membrane, consequently triggering mitophagy. Additionally, knockdown of FTMT, as well as deferiprone treatment, reduces ROS levels. In our study, after treatment with TSC-34, we observed a significant increase in FTMT expression, which can lead to changes in mitochondrial membrane polarity, resulting in mitochondrial damage and increased ROS production. These changes can disrupt mitochondrial morphology [37]. One of the main factors maintaining mitochondrial architecture is the mitochondrial contact site and cristae organizing system (MICOS) complex, which physically interacts with FECH. Disruption of MICOS function leads to PPIX accumulation and reduced FECH activity [38]. The interplay between FTMT and FECH may explain the effectiveness of ALA-PDT after TSC-34 treatment in the HCT116 and MCF-7 cell lines. In the case of Hs683 cells, despite the increase in FTMT expression, we did not observe a decrease in FECH expression. However, the described mechanism may influence the strong phototoxic effect observed in this cell line.

**Figure 9:**
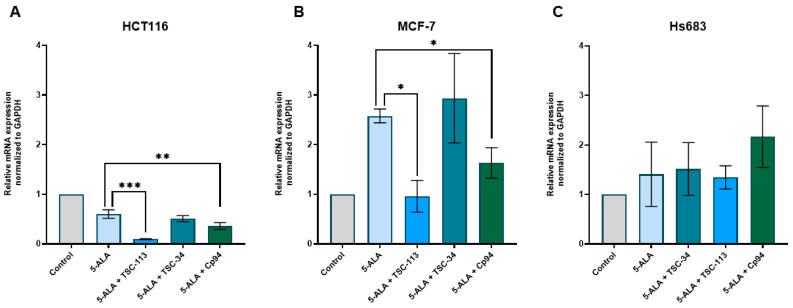
Expression of FXN in the (**A**): HCT116, (**B**): MCF-7, and (**C**): Hs683 cell lines. The data are presented as means ± standard deviation from three independent experiments and analyzed using one-way ANOVA with Tukey’s post hoc test, indicating significance levels as follows: * *p* < 0.05, ** *p* < 0.01, *** *p* < 0.001.

Our findings indicate a notable reduction in FNX expression (Figure 9A,B) when HCT116 and MCF-7 cell lines are treated with a combination of TSC-113 and Cp94 alongside 5-ALA. However, TSC-34 treatment did not result in significant changes. In contrast, no statistically significant alterations in gene expression were observed in the Hs683 cell line (Figure 9C). While FXN does not directly impact the heme biosynthetic pathway, studies conducted on human embryonic kidney cells indicate that diminished FXN expression leads to a gradual reduction in FECH levels over an extended period [39]. Primary symptoms associated with reduced FXN expression, such as decreased iron-sulfur cluster concentrations and indications of oxidative stress, manifest at an early stage. It has been proposed that the inhibition of FECH activity in these cells is attributed to a decline in the concentration of [2Fe-2S] clusters, crucial for the proper functioning of FECH [40]. Explaining the impact of alterations in FXN expression on ALA-PDT efficiency proves challenging due to the incomplete understanding of FXN’s role in cellular iron metabolism. Nonetheless, the reduced expression observed after treatment with TSC-113 in combination with 5-ALA in the HCT116 and MCF-7 cell lines suggests a potential connection between ALA-PDT efficiency and decreased FXN expression, possibly through an indirect modulation of FECH activity. Conversely, in cells treated with Cp94 in combination with 5-ALA, despite a decrease in FXN expression, this effect is not evident. Meanwhile, the absence of significant changes in the Hs683 cell line and the pronounced phototoxic effect following treatment with TSC-113 and TSC-34 indicate the involvement of other mechanisms independent of FXN in influencing the efficacy of treatment with these chelators. Interestingly, studies have demonstrated that in conditions of iron deficiency, FECH can dissociate an iron ion from heme, resulting in the formation of PPIX [41,42].

**Figure 10:**
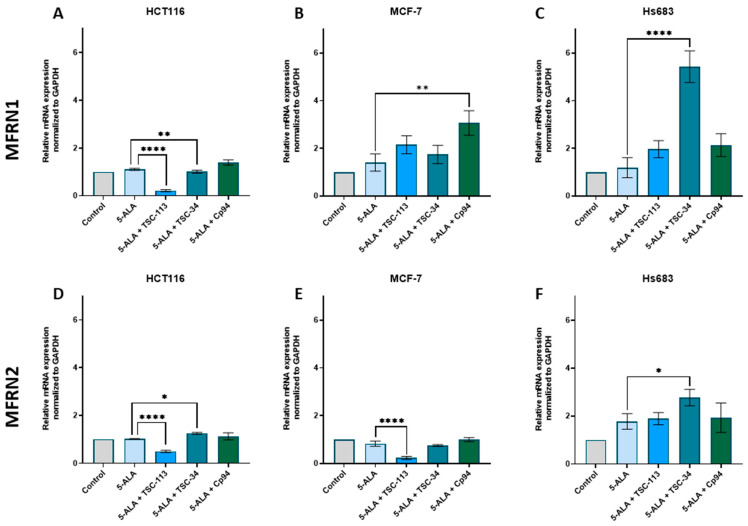
Expression of MFRN 1 and 2 genes on the (**A**,**D**): HCT116, (**B**,**E**): MCF-7 and (**C**,**F**): Hs683 cell lines. The data are presented as means ± standard deviation from three independent experiments and analyzed using one-way ANOVA with Tukey’s post hoc test, indicating significance levels as follows: * *p* < 0.05, ** *p* < 0.01, **** *p* < 0.0001.

The conducted research indicates that treatment with TSC-113 and 5-ALA results in a decrease in the expression of both forms of MFRNs on the HCT116 cell line (Figure 10A,D) and the MCF-7 cell line for MFRN2 (Figure 10E), while no significant changes in MRFN1 expression were observed. Treating the HCT116 cell line with the combination of TSC-34 and 5-ALA led to a slight increase in MFRN2 expression (Figure 10D). However, a notable increase in MFRN1 expression was observed after Cp94 treatment on the HCT116 and MCF7 cell lines (Figure 10A,B), while no changes were detected in MFRN2 expression (Figure 10D,E). In the Hs683 cell line, only TSC-34 treatment increased the expression of both MFRN1 and 2 (Figure 10C,F); in other cases, no significant changes were observed.

As mentioned above, MFRN is thought to form a complex with FECH and likely transfers an iron ion directly to the active center of FECH. Our observed decrease in the expression of both forms after treatment with TSC-113 in the HCT116 cell line and isoform 2 in the MCF-7 cell line may explain the efficacy of this derivative in ALA-PDT. The reduction in MFRN expression leads to a lack of iron transport necessary for heme biosynthesis, resulting in increased PPIX accumulation. This is consistent with the observation that the MCF-7 cell line, which has reduced MFRN1/2 expression, shows increased PPIX accumulation and a stronger phototoxic effect compared to healthy cells with higher MFRN1/2 expression [43]. Additionally, they showed that treating these cells with iron does not diminish the accumulation of PPIX, which proves that the expression of enzymes involved in heme biosynthesis is crucial. Another study demonstrated that cells overexpressing MFRN1 also exhibited increased glutathione levels, which promotes cell survival [44]. In our study, we observed an increased expression of MFRN1/2 in the Hs683 cell line following TSC-34 treatment, which should result in a weakened photodynamic effect. However, we did not observe this effect in our study. Nonetheless, it is important to note that this increase in MFRN1/2 expression could potentially be a response to the demand for iron ions and may not directly impact the efficacy of ALA-PDT.

**Figure 11:**
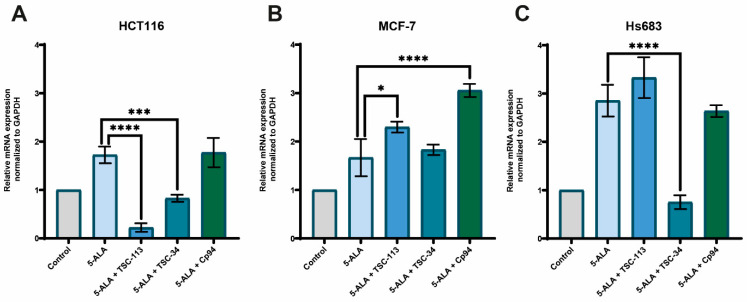
Expression of DMT1 in the (**A**): HCT116, (**B**): MCF-7, and (**C**): Hs683 cell lines. The data are presented as means ± standard deviation from three independent experiments and analyzed using one-way ANOVA with Tukey’s post hoc test, indicating significance levels as follows: * *p* < 0.05, *** *p* < 0.001, **** *p* < 0.0001.

Our findings revealed a significant increase in DMT1 expression following 5-ALA-only treatment across all tested cell lines compared to control groups (Figure 11). The observed increase in DMT1 expression after treatment with 5-ALA alone indicates an enhanced demand for iron ions. In our previous studies, we showed that cell lines treated with 5-ALA alone exhibited increased levels of free heme compared to control groups. This observation was considered a potential explanation for the absence of phototoxic effects in cells treated with 5-ALA alone [23] and is seemingly supported by DMT1 expression.

When chelators are combined with 5-ALA, a distinct pattern of DMT1 expression emerges. Specifically, in the HCT116 cell line, there is a significant decrease in DMT1 expression following treatment with TSC-34 and TSC-113 (Figure 11A). Conversely, treatment with Cp94 maintains DMT1 expression at a level comparable to that observed in cells treated with only 5-ALA. In the MCF-7 cell line, a significant increase in DMT1 expression is noted when treated with a combination of TSC-113 and Cp94 alongside 5-ALA (Figure 11B). Interestingly, no significant changes are observed for cells treated with TSC-34, although its expression remains comparable to that induced by 5-ALA alone. On the other hand, the Hs683 cell line exhibits a significant decrease in DMT1 expression when treated with TSC-34, while its expression level is otherwise similar to that of cells treated only with 5-ALA (Figure 11C). While the findings presented above may initially seem contradictory, existing literature highlights that different chelators can elicit distinct effects on DMT1 expression. For instance, studies demonstrate that treating rat lung cancer cells with quercetin leads to a decrease in DMT1 expression [18], whereas breast cancer cells subjected to DFO treatment exhibit the opposite effect, resulting in an increase in DMT1 expression [45]. Additionally, there is limited information on the regulation of DMT1 expression, particularly during disruptions of the iron availability in the cells, posing a challenge in correlating the obtained results with the efficiency of ALA-PDT. Nevertheless, a diminished expression of DMT1 may potentially enhance its efficiency by reducing iron uptake.

**Figure 12:**
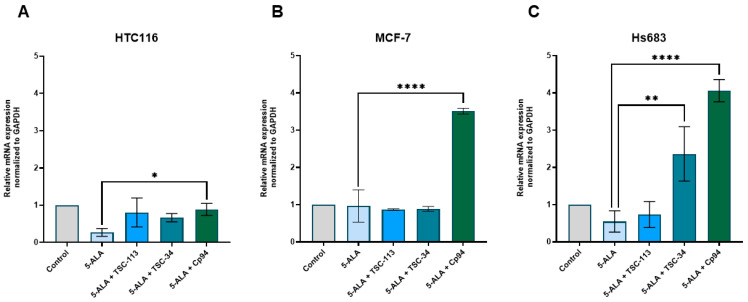
Expression of ABCB8 in the (**A**): HCT116, (**B**): MCF-7, and (**C**): Hs683 cell lines. The data are presented as means ± standard deviation from three independent experiments and analyzed using one-way ANOVA with Tukey’s post hoc test, indicating significance levels as follows: * *p* < 0.05, ** *p* < 0.01, **** *p* < 0.0001.

The results suggest that treatment with 5-ALA or the combination of TSC-113 and TSC-34 with 5-ALA does not significantly alter ABCB8 transcript levels in HCT116 and MCF-7 cell lines (Figure 12A,B). Notably, only the co-administration of Cp94 with 5-ALA induces a substantial increase in ABCB8 expression across all tested cell lines. However, in the Hs683 cell line, a significant increase in ABCB8 expression is observed not only following Cp94 and 5-ALA treatment but also with TSC-34 (Figure 12C). The impact of ABCB8 expression on the efficacy of ALA-PDT remains unexplored in existing literature, to the best of our knowledge. However, previous studies indicate that downregulation of the ABCB8 gene in renal cell carcinoma lines results in diminished cell survival, as evidenced by mitochondrial damage and heightened accumulation of ROS within mitochondria [21]. A comparable scenario was observed in mouse cardiomyocytes with reduced ABCB8 expression, leading to cardiomyopathy and mitochondrial iron accumulation [46]. This would elucidate the absence of a phototoxic effect following treatment with the combination of Cp94 and 5-ALA, positing that heightened expression of this transporter is linked to increased cell survival. However, in the case of TSC-34, despite an increase in ABCG8 expression, we still observed a phototoxic effect. This is consistent with our previous results for another transporter responsible for iron ejection from the cell, ABCG2. These results demonstrate the high efficacy and versatility of our compounds, particularly TSC-34, in enhancing the efficacy of ALA-PDT.

## 3. Materials and Methods

### 3.1. Synthesis–Reagents and Equipment

The chemical reagents employed in this study were acquired from Sigma-Aldrich (Burlington, MA, USA) and ACROS Organics (Thermo Fisher Scientific, Waltham, MA, USA). Thin layer chromatography (TLC) was conducted on alumina-coated silica gel 40 F254 plates (Merck, Drmstadt, Germany). Visualization of the chromatographic plates was achieved using ultraviolet light (254 nm) and subsequent exposure to iodine vapor for detection. Melting points were ascertained using an Optimelt MPA100 (Stanford Research Systems, Sunnyvale, CA, USA). Nuclear magnetic resonance (NMR) spectroscopy was used for the characterization of all synthesized compounds and was performed on a Bruker AM-400 spectrometer (399.95 MHz for ^1^H; 99.99 MHz for ^13^C; BrukerBioSpin Corp., Coventry, UK). Chemical shifts are delineated in parts per million (ppm), referenced to the internal standard tetramethylsilane-Si(CH_3_)_4_. Signals susceptible to rapid exchange were excluded when broad or indistinct. The syntheses of the studied compounds were performed on a CEM-DISCOVERY microwave reactor (CEM Corporation, Matthews, NC, USA) with temperature and pressure control. The mass spectrometry characterizations of synthesized TSCs were collected using an electrospray single-quad Agilent Infinity Lab LC/MSDXT mass spectrometer, equipped with an Agilent HPLC 1260 Infinity II system (Agilent Technologies, Santa Clara, CA, USA) and SB C18 column (1.8 μm, 2.1 × 50 mm). Logarithmic partition coefficients (cLogP) were computationally determined by employing ChemDraw version 21 (Perkin-Elmer, Waltham, MA, USA).

### 3.2. General Synthetic Protocol for the Creation of Aromatic TSCs

In brief, in a 25 mL sealed tube equipped with a magnetic stirrer, equimolar amounts of (1,1′-thiocarbonyl)bis-*1H*-imidazole (1 eq) and an appropriate *N*-alkyl or *N*-aryl derivatives of piperazine or thiomorpholine (1 eq) were added, followed by the addition of 12 mL of methylene chloride. The system was then tightly sealed with a septum cap and stirred at room temperature for 24 h. The resulting intermediate was extracted three times with distilled water. The organic phase obtained was dried over anhydrous calcium chloride and then evaporated under reduced pressure using a rotary evaporator. To the resulting imidazolylthioketone derivative (1 eq), an equimolar amount of hydrazine hydrate and 25 mL of ethanol were added. The reaction mixture was heated for 2 h under reflux. Then, the reaction mixture was stored in a laboratory refrigerator at −20 °C, resulting in the formation of a crystalline solid, which was subsequently crystallized from anhydrous methanol to form the desired TSC (Figure S1 in Supporting Information).

In a 10 mL glass vessel equipped with a magnetic stirrer, equimolar amounts of TSC and the corresponding aldehyde were added. Subsequently, 5 mL of dry ethanol and three drops of ice-cold acetic acid were added. The vessel was hermetically sealed with a cap and positioned within a CEM microwave reactor at 83 °C for 20 min (the power of the reactor did not exceed 100 W). The resultant product was crystallized using methanol. The spectral characterization of all synthesized aromatic TSCs is presented in the Supporting Information.

### 3.3. Cell Lines and Cell Culture

The HCT116 (colorectal cancer) and MCF-7 (breast cancer) cell lines were procured from the American Type Culture Collection (ATCC; Manassas, VA, USA), while the Hs683 cell line was generously provided by Prof. Gabriela Kramer-Marek from the Institute of Cancer Research (London, UK). These cell lines were cultured in Dulbecco’s Modified Eagle’s Medium (DMEM; Sigma-Aldrich; St. Louis, MO, USA) supplemented with 10% fetal bovine serum (FBS; Sigma-Aldrich; St. Louis, MO, USA) and 1% antibiotic mixture (penicillin and streptomycin; Gibco; Grand Island, NY, USA). Culturing was conducted in 75 cm^2^ flasks (Nunc, Sigma-Aldrich; St. Louis, MO, USA) at 37 °C in a humidified atmosphere containing 5% CO_2_. Before experimentation, all cell lines underwent mycoplasma contamination via PCR.

### 3.4. Cytotoxicity Studies

Cells were seeded at a density of 5000 cells per well in a 96-well plate and incubated for 24 h under standard conditions. Subsequently, the tested compounds were added at a concentration of 50 µM and incubated for 72 h. Following this incubation period, the medium containing the test compounds was aspirated, and phenol red-free medium supplemented with CellTiter 96^®^AQueous One Solution (MTS, Promega, Madison, WI, USA) was added. After approximately 1 h, the absorbance at a wavelength of 490 nm was measured using a multi-well plate reader (Synergy 4, BioTek, Winooski, VT, USA). The obtained absorbance values were utilized to calculate the antiproliferative activity of the tested compounds.

### 3.5. Chelating Abilities

The compounds, along with iron (III) ions, were initially dissolved in dimethylsulfoxide (DMSO) to yield a stock solution of 8.358 mM. Subsequently, all samples were diluted with ultrapure water to obtain a concentration of 100 μM. Solutions containing the tested compounds and iron ions were then prepared by transferring appropriate volumes into a 96-well black plate with a transparent bottom (Nunc, Sigma-Aldrich; St. Louis, MO, USA). Each well was loaded with 200 μL of solution, comprising the tested compound at a concentration of 50 μM and increasing concentrations of iron ions from 0 to 50 μM. Absorption spectra were recorded following a 4 h incubation at room temperature using a Varioskan LUX (Thermo Fisher Scientific, Waltham, MA, USA) multi-well plate reader in the 275–800 nm range with a 5 nm step.

### 3.6. Accumulation of PPIX

Cells were seeded into a 96-well black plate with a transparent bottom (Nunc, Sigma-Aldrich; St. Louis, MO, USA) at a density of 1.1 × 10^4^ cells per well and cultured under standard conditions for 24 h. After incubation, the cells were treated with TSC and Cp94 at a concentration of 25 μM, 1 mM of freshly prepared 5-ALA, and their combination in a phenol red-free medium. After 24 h of incubation with the compounds, PPIX fluorescence was measured using a multi-well plate reader (Synergy 4, BioTek, VT, USA) at an excitation wavelength of 407 nm and an emission wavelength of 638 nm. The obtained fluorescence values were then used to calculate the relative fluorescence intensity of PPIX as a percentage of 5-ALA. All experiments were conducted under reduced illumination to prevent photobleaching of PPIX and were repeated four times.

### 3.7. Phototoxic Effect

Cells were seeded and treated following the same procedure as described for measuring PPIX accumulation. Following a 24 h incubation with the compounds, the cells underwent three washes with phenol red-free medium and were then exposed to red light (634 nm ± 5 nm) at a dose of 12 J/cm^2^. Following irradiation, fresh medium was replenished, and the cells were further incubated under standard conditions for an additional 24 h. Simultaneously, a control experiment was conducted without irradiation. Cell viability was assessed using the MTS assay, following the same protocol as for cytotoxicity evaluation. All experiments were carried out in the absence of light. The results are presented as the mean ± standard deviation of three independent experiments, and cell viability was calculated using GraphPad Prism 8 software (GraphPad Software Inc.; San Diego, CA, USA).

### 3.8. Genes Expression

Cells were seeded at a density of 4 × 10^5^ cells per dish (3 cm plastic Petri dishes; Nunc, Sigma-Aldrich; St. Louis, MO, USA) and incubated for 24 h at 37 °C. Subsequently, the old medium was aspirated, and solutions of 5-ALA (1 mM), TSC, and Cp94 (25 µM) were added. After an additional 24 h of incubation, total RNA was extracted from the cells using TRIzol reagent (Invitrogen; Carlsbad, CA, USA) following the manufacturer’s protocol.

For reverse transcription, 2 µg of total RNA was utilized with the GoScript™ Reverse Transcriptase kit (Promega; Madison, WI, USA) and Oligo(dT)23 Primers (Sigma-Aldrich; St. Louis, MO, USA). Quantitative RT-PCR was performed using a CFX96 Touch™ Real-Time PCR Detection System (Biorad; Hercules, CA, USA), SYBR^®^ Green Master Mix (Biorad; Hercules, CA, USA), the appropriate primer pairs, and cDNA template. Primer sequences were designed using Primer3 online software, https://www.ncbi.nlm.nih.gov/tools/primer-blast/ accessed on 20 March 2023 (Table 3) and synthesized and provided by Sigma-Aldrich (Sigma-Aldrich; St. Louis, MO, USA). Data analysis involved comparing the expression of the target gene to a reference gene, GAPDH, using the 2^−∆∆CT^ method (Livak and Schmittgen method). The experiments were conducted with a minimum of three repetitions in triplicate.

### 3.9. Statistical Analysis

All experiments were repeated at least three times independently, with the results presented as the mean ± standard deviation. Data analysis was performed using GraphPad Prism 8 software (GraphPad Software Inc., San Diego, CA, USA). Statistical significance between groups was assessed using one-way or two-way ANOVA followed by Tukey’s post hoc test. A two-tailed *p*-value of 0.05 or less was considered statistically significant, with significance levels indicated as follows: * *p* < 0.05, ** *p* < 0.01, *** *p* < 0.001, **** *p* < 0.0001.

## 4. Conclusions

This paper presents an analysis of the effects of selected derivatives from the TSC group on iron metabolism in ALA-PDT. The compounds were selected based on our previous studies, taking into account their ability to increase PPIX levels after incubation with 5-ALA while maintaining the absence of dark toxicity. The effectiveness of our novel TSC derivatives in increasing PPIX concentrations is greater than commonly used iron chelators. The main mechanism of the observed effect is related to the formation of iron complexes. Therefore, this ability was confirmed by the spectroscopic studies in which isosbestic points indicative of the generation of complexes with iron were measured.

It is also worth mentioning that our compounds inhibit the proliferation of cancer cells under red light irradiation. This shows that the concentration used has a therapeutic effect, which is extremely important from a practical application point of view. Low doses are desirable to minimize side effects. In contrast to our TSC derivatives, the Cp94 reference used was not effective at the concentration tested.

An expression analysis of genes involved in iron metabolism included both common targets and those for which there is little information. The standard gene studied in the context of PPIX accumulation is FECH. Our study confirmed the widely held opinion that a reduction in FECH expression has a positive effect on PPIX accumulation for the HCT116 and MCF-7 cell lines. However, our study of the Hs683 line, which exhibits non-standard behavior, once again overturned the widely held trend. Namely, for the TSC-34 derivative, despite an increase in FECH, we registered both an increase in PPIX and a marked decrease in cell viability after irradiation. We observed a similar trend for HO-1, which was consistent with the literature for the HCT116 and MCF-7 cell lines and a break from this trend for Hs683 cells. The increase in HO-1 expression is a non-standard phenomenon in the context of increased PPIX accumulation and phototoxic effects after incubation with TSC-34. In the case of FTMT, the expression of which was increased in the majority of cases, we observed an interesting correlation with FECH. Namely, we hypothesize that an increase in FTMT expression results in the destruction of MICOS through the production of ROS, which has a positive effect on increasing the concentration of PPIX and the effect of PDT. We also recorded interaction with FECH for downregulated FXN but in a less direct way; in this case, it is related to the decrease in iron-sulfur cluster concentrations affecting the concentration of FECH. MFRN, as the main supplier of iron ions to FECH, also showed a correlation; namely, its decrease was the explanation for the induced photocytotoxic effect for TSC-113. An explanation for this effect could also be the downregulation of the iron ion transporter, DMT1, and expression for the HCT116 and Hs683 cell lines. The last gene analyzed was ABCG8, which is responsible for iron efflux from the cell. Therefore, in our study, we traced the fate of iron from entry to exit of the cell. The results indicate that, despite overexpression of this gene occurring in the Hs683 cell line, TSC-34 increased the concentration of PPIX to the extent that it was able to induce an effect after irradiation. These results demonstrate the great potential of our compounds, in particular the TSC-34 derivative, in increasing the efficacy of ALA-PDT.

## Figures and Tables

**Table 1:** Antiproliferative properties expressed as IC_50_ of the tested TSCs and Cp94 on a panel of tumor cell lines and human normal fibroblasts (NHDFs).

No.	TSC	IC_50_ [µM]			
		HCT116	MCF-7	Hs683	NHDF
1	TSC-34	18.31 ± 0.92	>50	>50	13.06 ± 1.90
2	TSC-82	47.56 ± 1.99	>50	>50	>50
3	TSC-102	>50	>50	>50	>50
4	TSC-109	30.87 ± 0.64	42.97 ± 1.33	>50	>50
5	TSC-113	>50	>50	>50	>50
6	TSC-116	34.88 ± 0.96	>50	>50	>50
7	TSC-140	>50	>50	>50	>50
8	TSC-142	>50	>50	>50	>50
9	TSC-145	>50	>50	>50	>50
10	TSC-146	>50	>50	>50	>50
11	TSC-147	>50	>50	>50	>50
12	TSC-197	>50	>50	>50	>50
13	Cp94	>50	>50	>50	>50

**Table 2:** Spectroscopic data for tested compounds dissolved in water with iron (III) ions (Fe^3+^).

Sample	Compound	Isosbestic Point [nm]	Complex with Fe^3+^
	λ_max_ [nm]		λ_max_ [nm]
Cp-94	280	290	510
TSC-34	337	366	415
TSC-109	340	378	449
TSC-113	305	342	362
TSC-116	308	345	385

**Table 3:** Primers designed and used in this study.

Gene Name	Sequence 5→3 (F—Forward, R—Reverse)	
*DMT1*	F	GGTCACGCTTTGCCCGA
	R	CAATCCGCCAGCCTAGTCC
*MFRN1*	F	ACTCGGTGAAGACACGAATGC
	R	CAGCTATCCCGTTGGCTAGG
*MFRN2*	F	CACTGCGTGATGTACCCCAT
	R	CAACACATTGCGATAGCGGG
*ABCB8*	F	TATTTCGGGTCGGGATTCGG
	R	TCCAGTTTTCCCATTACGCCA
*FNX*	F	TGGACCTAAGCGTTATGACTGG
	R	TCTTCCCGTGTGAGTTGCTT
*FTMT*	F	CCATCAACCGCCAGATCAAC
	R	GTGCAATTCCAGCAACGACT
*FECH*	F	GATGAATTGTCCCCCAACAC
	R	GCTTCCGTCCCACTTGATTA
*HO-1*	F	CATCCCCTACACACCAGCCA
	R	ATGTTGGGGAAGGTGAAGAAGG

## Data Availability

The data presented in this study are available on request from the corresponding author (anna.mrozek-wilczkiewicz@us.edu.pl or anna.mrozek-wilczkiewicz@polsl.pl).

## Supplementary Materials

The following supporting information can be downloaded at: https://www.mdpi.com/article/10.3390/ijms251910468/s1.
