# Supplementary material for: Iron Metabolism in Aminolevulinic Acid-Photodynamic Therapy with Iron Chelators from the Thiosemicarbazone Group
Source: Int J Mol Sci. 2024 Sep 28;25(19):10468. doi: 10.3390/ijms251910468 (PMC11476630; doi:10.3390/ijms251910468)
Supplement: Supplementary file 1 [file ijms-25-10468-s001.zip › ijms-3193395-supplementary.pdf]

Iron Metabolism in Aminolevulinic Acid-Photodynamic Therapy with Iron Chelators from the Thiosemicarbazone Group

Robert Gawecki [1,2], Patrycja Rawicka [1], Marta Rogalska [3], Maciej Serda [3] and Anna Mrozek-Wilczkiewicz [1,4,*]

[1] Institute of Physics, University of Silesia in Katowice, 75 Pułku Piechoty 1A, 41-500 Chorzow, Poland;
robert.gawecki@us.edu.pl (R.G.); patrycja.rawicka@us.edu.pl (P.R.)
[2] SPIN-Lab Centre for Microscopic Research of Matter, University of Silesia in Katowice, 75 Pułku Piechoty
1A, 41-500 Chorzow, Poland
[3] Institute of Chemistry, University of Silesia in Katowice, Szkolna 9, 40-006 Katowice, Poland;
rejmund.m@gmail.com (M.R.); maciej.serda@us.edu.pl (M.S.)
[4] Department of Systems Biology and Engineering, Silesian University of Technology, Akademicka 16,
44-100 Gliwice, Poland
* Correspondence: anna.mrozek-wilczkiewicz@us.edu.pl or anna.mrozek-wilczkiewicz@polsl.pl

SUPPORTING INFORMATION

1. ^{1}H- and ^{13}C-NMR spectral characterization of synthesized thiosemicarbazides and thiosemicarbazones.
2. Absorption spectrum obtained for the solvent used in titration measurements (sample blank).

1.

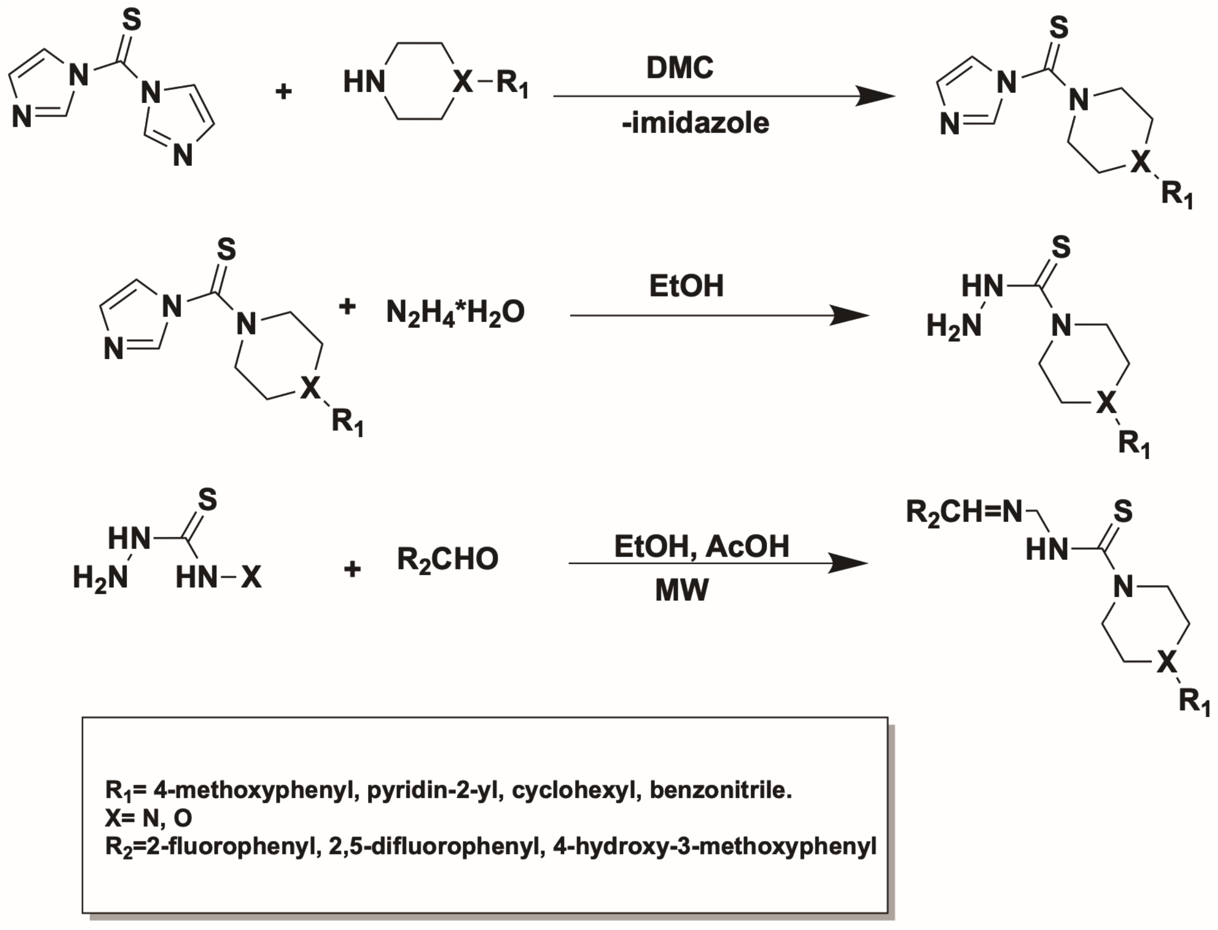

Figure S1. The synthetic protocol used for obtaining selected thiosemicarbazones.

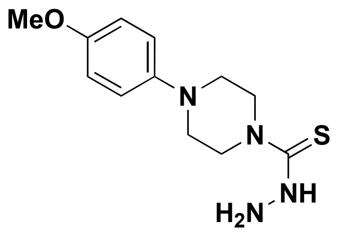

4-(4-methoxyphenyl)piperazine-1-carbothiohydrazide

^{1}H-NMR (400 MHz, *d_6*-DMSO, ppm): δ 3.00 (m, 3H, CH_3), 3.86 (m, 4H, CH_2), 4.11 (m, 4H, CH_2), 4.77 (s, 2H, NH_2), 6.83 (d, 2H, J = 9.0 Hz), 6.90 (m, 2H, ArH), 9.17 (s, 1H, NH).
^{13}C-NMR (101 MHz, *d_6*-DMSO, ppm): δ 19.0; 49.9; 56.5; 114.8; 118.2; 145.4; 153.7; 183.1.
LogP: 0.71
Yield: 81%.

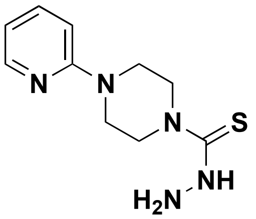

4-(pyridin-2-yl)piperazine-1-carbothiohydrazide

^{1}H-NMR (400 MHz, *d_6*-DMSO, ppm): δ 3.18 (s, 4H, CH_2), 3.85 (m, 4H, CH_2), 4.77 (s, 2H, NH_2), 6.67 (m, 1H, Ar-H), 6.83 (m, 1H, Ar-H), 7.56 (m, 1H, Ar-H), 8.13 (m, 1H, Ar-H), 9.13 (s, 1H, NH).

^{13}C-NMR (101 MHz, *d_6*-DMSO, ppm): δ 44.4; 47.2; 107.6; 113.7; 138.0; 148.0; 159.1; 183.0.

LogP: -0.02

Yield: 98 %.

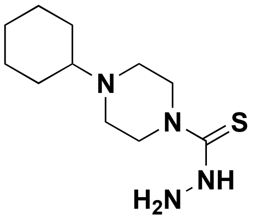

4-cyclohexylpiperazine-1-carbothiohydrazide

^{1}H-NMR (400 MHz, *d_6*-DMSO, ppm): δ 1.19 (m, 6H, CH_2), 1.73 (d, 5H, J = 8.4 Hz), 3.36 (m, 4H, CH_2), 3.66 (s, 4H, CH_2), 4.73 (s, 2H, NH_2), 9.02 (s, 1H, NH).

^{13}C-NMR (101 MHz, *d_6*-DMSO, ppm): δ 25.7; 26.3; 28.7; 48.3; 48.7; 62.9; 182.9.

LogP: 1.65

Yield: 59 %.

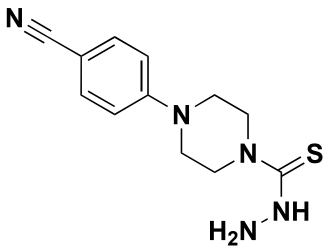

4-(4-cyanophenyl)piperazine-1-carbothiohydrazide

^{1}H-NMR (400 MHz, *d_6*-DMSO, ppm): δ 3.42 (m, 4H, CH_2), 3.89 (m, 4H, CH_2), 4.78 (s, 2H, NH_2), 7.05 (s, 2H, Ar-H), 7.60 (m, 2H, Ar-H), 9.15 (s, 1H, NH).

^{13}C-NMR (101 MHz, *d_6*-DMSO, ppm): δ 45.9; 48.9; 98.6; 114.3; 120.5; 133.8; 153.0; 182.9.

LogP: 0.78

Yield: 70 %.

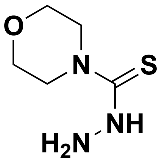

morpholine-4-carbothiohydrazide

^{1}H-NMR (400 MHz, *d_6*-DMSO, ppm): δ 3.59 (m, 4H, CH_2), 3.68 (m, 4H, CH_2), 4.79 (s, 2H, NH_2), 9.12 (s, 1H, NH).

LogP: -0.84

Yield: 74 %.

TSC-82

(*E*)-*N*'-(2-fluorobenzylidene)-4-(4-methoxyphenyl)piperazine-1-carbothiohydrazide

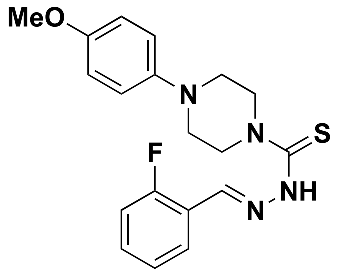

^{1}H-NMR (400 MHz, *d$_6$*-DMSO, ppm): δ 3.13 (bs, 4H, CH_2), 3.70 (s, 3H, -OCH_3), 4.07 (bs, 4H, CH_2), 6.85 (d, 2H, J = 8.9 Hz), 6.95 (d, 2H, J = 9.0 Hz), 7.29 (dd, J_1= 13.1 Hz, J_2=5.3 Hz, 2H), 7.46 (dd, J_1= 13.6 Hz, J_2= 6.1 Hz, 1H), 7.85 (t, 1H, J = 7.5 Hz), 8.40 (s, 1H, CH), 11.38 (s, 1H, NH).

^{13}C-NMR (126 MHz, *d$_6$*-DMSO, ppm): δ 181.0, 162.1, 160.1, 153.7, 145.4, 137.07 (d, J= 4.5 Hz), 132.1, 132.0, 126.67, 126.65, 125.4 (d, J= 3.3 Hz), 122.4 (d, J= 9.9 Hz), 118.2, 116.6, 116.4, 114.8, 55.64, 50.35, 50.17.

LogP: 2.67

Yield: 30%.

TSC-102

(*E*)-*N*'-(2-fluorobenzylidene)-4-(pyridin-2-yl)piperazine-1-carbothiohydrazide

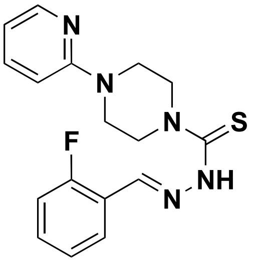

^{1}H-NMR (400 MHz, *d$_6$*-DMSO, ppm): δ 3.65 (bs, 4H, CH_2), 4.07 (bs, 4H, CH_2), 6.68 (t, 1H, J= 5.5 Hz), 6.85 (d, 1H, J= 8.5 Hz), 7.28 (t, 2H, J= 9.2 Hz), 7.46 (d, 1H, J= 6.3 Hz), 7.57 (t, 1H, J=7.7 Hz), 7.86 (t, 1H, J=7.4 Hz), 8.15 (s, 1H, ArH), 8.41 (s, 1H, CH), 11.39 (bs, 1H, NH).

^{13}C-NMR (126 MHz, *d$_6$*-DMSO, ppm): 181.0, 162.1, 160.1, 159.0, 148.0, 138.1, 137.1 (d, J= 4.3 Hz), 132.1, 132.0, 126.7, 125.4 (d, J= 3.2 Hz), 122.4 (d, J= 9.9 Hz), 116.6, 116.4, 113.7, 107.5, 50.0, 44.6.

LogP: 1.94

Yield: 62%

TSC-109

(*E*)-4-cyclohexyl-*N*'-(2-fluorobenzylidene)piperazine-1-carbothiohydrazide

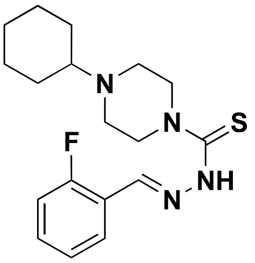

^{1}H-NMR (400 MHz, d_6-DMSO, ppm): δ 1.20 (t, 4H, *J*= 9.8 Hz), 1.58 (d, 1H, *J*= 11.6 Hz), 1.71-1.81 (m, 4H, CH_2), 2.27 (s, 1H, CH_2), 2.58 (s, 4H, CH_2), 3.89 (s, 4H, CH_2), 4.09 (m, 1H, CH_2), 7.27 (t, 2H, *J*= 8.6 Hz), 7.45 (dd, 1H, J_1= 13.6 Hz, J_2= 6.0 Hz), 7.83 (t, 1H, *J*= 7.3 Hz), 8.37 (s, 1H, CH), 11.25 (bs, 1H, NH).

^{13}C-NMR (126 MHz, d_6-DMSO, ppm): δ180.7, 162.0, 160.0, 136.8, 132.0 (d, *J*= 8.4 Hz), 126.6, 125.4 (d, *J*= 3.1 Hz), 122.4 (d, *J*= 9.9 Hz), 116.6, 116.4, 62.9, 50.8, 49.0, 31.2, 28.7, 26.3, 25.7.

LogP: 3.61

Yield: 19 %

TSC-113

(*E*)-4-(4-cyanophenyl)-*N*'-(2-fluorobenzylidene)piperazine-1-carbothiohydrazide

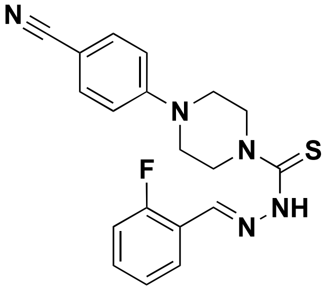

^{1}H-NMR (400 MHz, d_6-DMSO, ppm): δ 3.54 (m, 4H, CH_2), 4.09 (m, 4H, CH_2), 7.01 (d, 2H, *J*= 8.8 Hz), 7.28 (dd, J_1= 13.2 Hz, J_2= 6.9 Hz, 2H), 7.46 (dd, 1H, J_1 = 13.3 Hz, J_2 = 6.4 Hz), 7.61 (d, 2H, *J* = 8.8 Hz), 7.87 (t, 1H, *J* = 7.4 Hz), 8.41 (s, 1H, CH), 11.41 (bs, 1H, NH).

^{13}C-NMR (126 MHz, d_6-DMSO, ppm): δ 180.9, 162.1, 160.1, 153.1, 153.0, 137.2, 137.1, 133.8, 132.1 (d, *J*= 8.5 Hz) 126.7, 126.7, 125.4 (d, *J*= 3.1 Hz), 122.3 (d, *J*= 10 Hz), 120.5, 116.5, 116.4, 114.2, 98.5, 49.56, 49.07, 46.1

LogP: 2.74

Yield: 56 %

TSC-116

(*E*)-*N'*-(2-fluorobenzylidene)morpholine-4-carbothiohydrazide

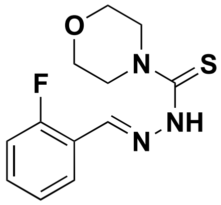

^{1}H-NMR (400 MHz, *d$_6$*-DMSO, ppm): δ 3.68 (m, 4H, CH_2), 3.93 (m, 4H, CH_2), 7.28 (dd, 2H, J_1= 13.0 Hz, J_2= 5.4 Hz, Ar-H), 7.46 (dd, 1H, J_1= 13.9 Hz, J_2= 6.7 Hz), 7.83 (t, 1H, J = 7.6 Hz), 8.38 (s, 1H, CH), 11.35 (bs, 1H, NH).

^{13}C-NMR (126 MHz, *d$_6$*-DMSO, ppm): δ 181.2, 162.0, 160.1, 137.2 (d, J= 4.4 Hz), 132.0 (d, J = 8.5 Hz), 126.7 (d, J= 2.6 Hz), 122.3, 116.5 (d, J= 20.7 Hz), 66.47, 51.01.

LogP: 1.12

Yield: 51 %

TSC-140

(*E*)-*N*-ethyl-2-(2-fluorobenzylidene)hydrazine-1-carbothioamide

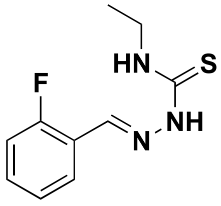

^{1}H-NMR (400 MHz, *d$_6$*-DMSO, ppm): δ 1.16 (t, J= 7.1 Hz, 3H, -CH_3), 3.60 (m, 2H, -CH_2), 7.26 (dd, 2H, J_1= 13.2 Hz, J_2= 5.7 Hz, ArH), 7.46 (dd, 1H, J_1= 13.5 Hz, J_2= 6.0 Hz, ArH), 8.23 (t, J= 7.2 Hz, 1H, ArH), 8.29 (s, 1H, CH), 8.62 (t, J= 5.5 Hz, 1H, NH), 11.56 (bs, 1H, NH).

^{13}C-NMR (126 MHz, *d$_6$*-DMSO, ppm): δ 177.2, 162.2, 160.2, 134.6 (d, J = 5.1 Hz), 132.1 (d, J = 8.5 Hz), 127.2 (d, J = 2.4 Hz), 125.1 (d, J = 3.1 Hz), 122.3 (d, J = 9.7 Hz), 116.4, 38.80, 15.02.

LogP: 2.71

Yield: 43 %

TSC142

(*E*)-*N*-benzyl-2-(2-fluorobenzylidene)hydrazine-1-carbothioamide

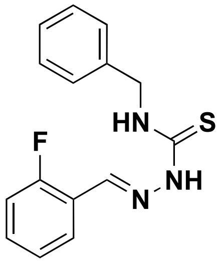

^{1}H-NMR (400 MHz, d_6-DMSO, ppm): δ 11.74 (bs, 1H, NH), 9.17 (t, J= 6.3 Hz, 1H, NH), 8.33 (s, 1H, CH), 8.26 (td, J_1 = 7.7, J_2= 1.7 Hz, 1H, ArH), 7.51 – 7.40 (m, 1H, ArH), 7.40–7.30 (m, 4H, ArH), 7.30 – 7.20 (m, 3H, ArH), 4.86 (d, J= 6.2 Hz, 2H, CH_2).

^{13}C-NMR (126 MHz, d_6-DMSO, ppm): δ 178.2, 162.3, 160.3, 139.8, 135.1 (d, J= 5.2 Hz), 132.2 (d, J= 8.4 Hz), 128.6, 127.6, 127.3 (m), 125.1 (d, J= 3.1 Hz), 122.30 (d, J= 9.7 Hz), 116.4, 116.2, 47.1.

LogP: 4.25

Yield: 64%

TSC-145

(*E*)-*N*-benzyl-2-(2,5-difluorobenzylidene)hydrazine-1-carbothioamide

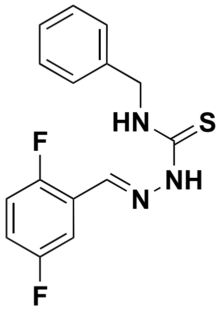

^{1}H-NMR (400 MHz, d_6-DMSO, ppm): δ 11.81 (bs, 1H, NH), 9.32 (t, J= 6.4 Hz, 1H, NH), 8.27 (s, 1H, CH), 8.17 (dt, J_1= 9.3 Hz, J_2= 4.1 Hz, 1H, ArH), 7.35 (m, 6H, ArH), 7.25 (s, 1H, ArH), 4.87 (d, J= 6.2 Hz, 2H, CH_2).

^{13}C-NMR (126 MHz, d_6-DMSO, ppm): δ 178.3, 160.0 (d, J= 1.8 Hz), 158.2 (d, J = 48.5 Hz), 139.7, 133.9 (t, J= 3.4 Hz), 128.6, 127.5, 127.2, 118.6 (dd, J_1= 25.2 Hz, J_2= 8.8 Hz), 118.1 (dd, J_1= 24.1 Hz, 8.9 Hz), 112.9 (d, J= 3.0 Hz), 112.7 (d, J= 3.0 Hz), 47.0.

LogP: 4.41

Yield: 24%

TSC-146

(*E*)-2-(2,6-difluorobenzylidene)-*N*-ethylhydrazine-1-carbothioamide

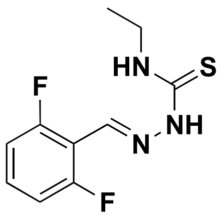

^{1}H-NMR (400 MHz, *d_6*-DMSO, ppm): δ 11.67 (bs,1H, NH), 8.21 (s, 1H, CH), 7.99 (s, 1H, NH), 7.54 – 7.44 (m, 1H, ArH), 7.19 (t, J = 8.6 Hz, 2H, ArH), 3.63 – 3.53 (m, 2H, -CH_2), 1.14 (t, J= 7.1 Hz, 3H, CH_3).

^{13}C-NMR (126 MHz, *d_6*-DMSO, ppm): δ 177.4, 162.0 (d, J= 6.8 Hz), 159.5 (d, J= 6.5 Hz), 132.6, 132.0 (t, J= 10.6 Hz), 112.8 (d, J= 5.3 Hz), 112.7 (dd, J_1 = 19.2 Hz, J_2= 5.3 Hz), 112.6 (d, J= 5.3 Hz), 111.9 (t, J= 14.0 Hz), 38.9, 14.8.

LogP: 2.87

Yield: 58 %

TSC-147

(*E*)-2-(2,6-difluorobenzylidene)-*N*-methylhydrazine-1-carbothioamide

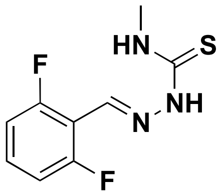

^{1}H-NMR (400 MHz, *d_6*-DMSO, ppm): δ 11.69 (bs, 1H, NH), 8.21 (s, 1H, CH), 8.03 (m, 1H, NH), 7.53–7.45 (m, 1H, ArH), 7.19 (t, J= 8.6 Hz, 2H, ArH), 3.02 (d, J= 4.3 Hz, 3H, CH_3).

^{13}C-NMR (126 MHz, *d_6*-DMSO, ppm): δ 178.3, 161.7 (d, J= 6.6 Hz), 159.7 (d, J= 6.7 Hz), 132.5, 132.0 (t, J= 10.6 Hz), 112.8 (d, J= 4.6 Hz), 112.6 (d, J= 4.5 Hz), 111.9 (t, J= 14.0 Hz), 31.5.

LogP: 2.53

Yield: 43 %

TSC-197

(*E*)-*N*-ethyl-2-(4-hydroxy-3-methoxybenzylidene)hydrazine-1-carbothioamide

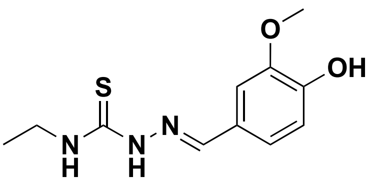

H-NMR (400 MHz, d_6-DMSO, ppm): δ 11.26 (s, 1H, NH), 9.46 (s, 1H, OH), 8.38 (t, *J*= 6.0 Hz, 1H, NH), 7.95 (s, 1H, CH), 7.38 (d, *J*= 1.9 Hz, 1H, ArH), 7.14 (dd, J_1 = 8.2 Hz, J_2=1.9 Hz, 1H, ArH), 6.80 (d, *J*= 8.2 Hz, 1H, ArH), 3.84 (s, 3H, -OCH_3), 3.66 – 3.55 (m, 2H, CH_2), 1.16 (t, *J*= 7.1 Hz, 3H, CH_3).

^{13}C-NMR (126 MHz, d_6-DMSO, ppm): δ 176.8, 149.2, 148.4, 143.1, 126.0, 122.3, 115.9, 110.6, 56.3, 38.7, 15.2.

LogP: 2.04

Yield: 68 %

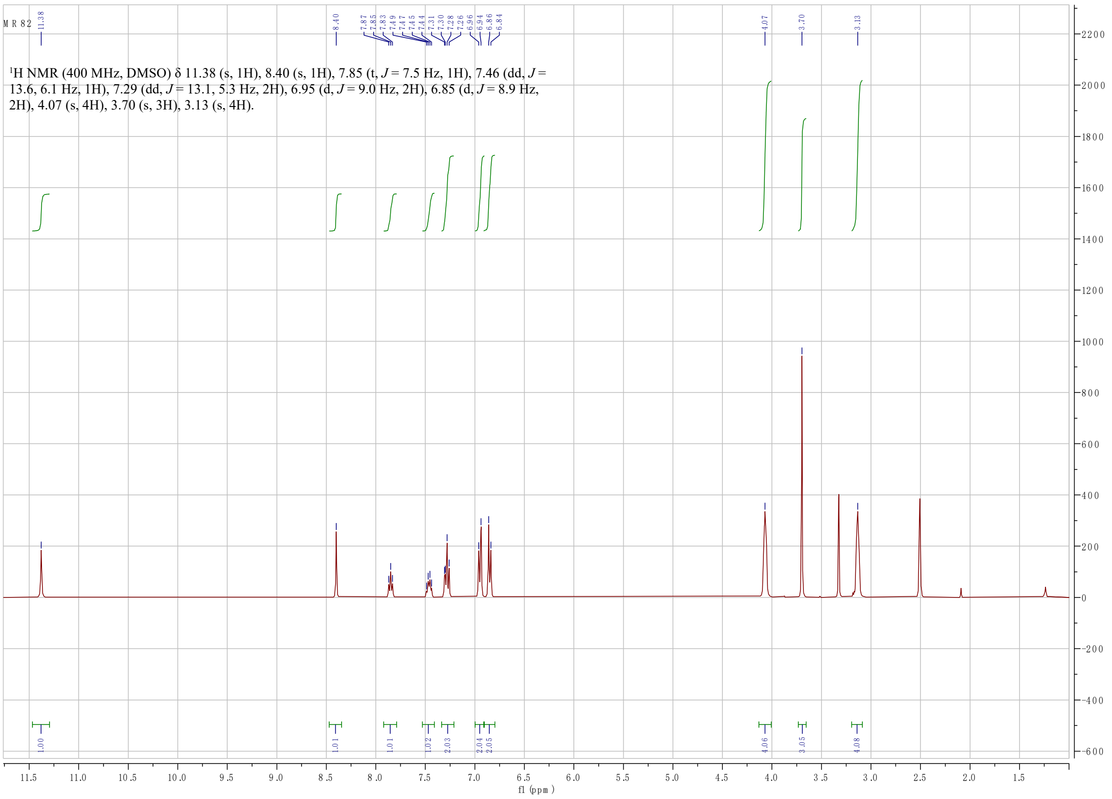

^{1}H-NMR spectrum of thiosemicarbazone TSC-82.

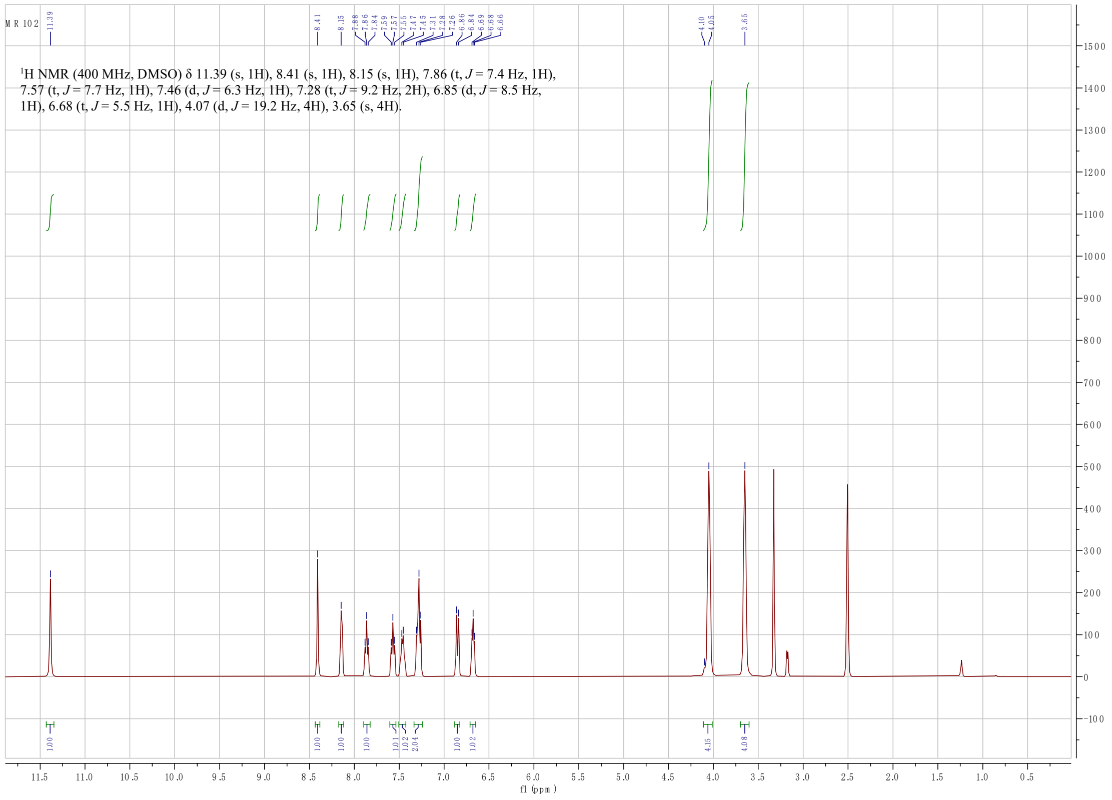

^{1}H-NMR spectrum of thiosemicarbazone TSC-102.

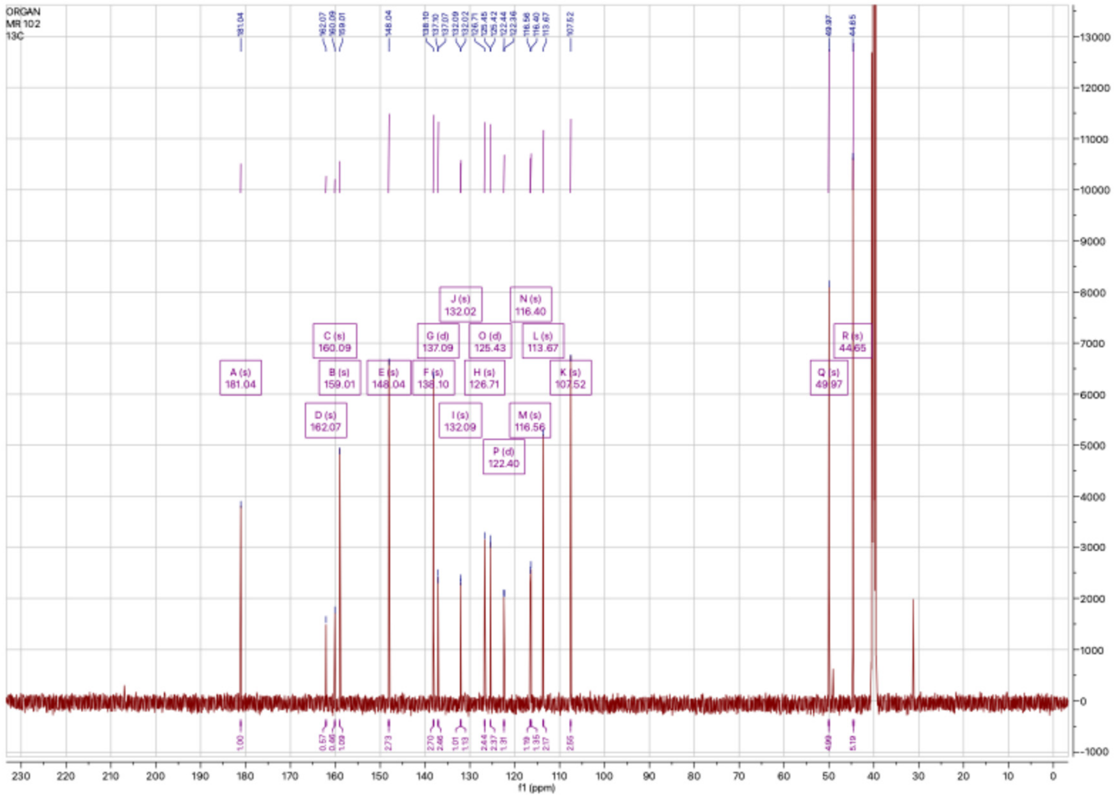

^{13}C-NMR spectrum of thiosemicarbazone TSC-102.

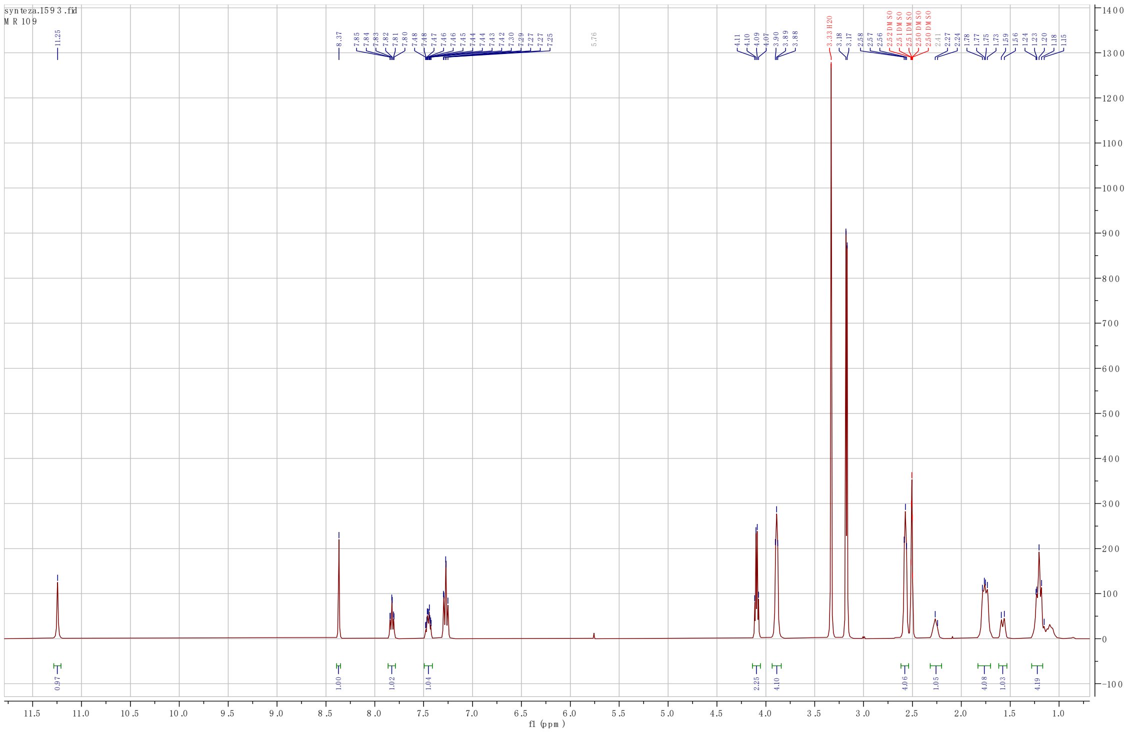

^{1}H-NMR spectrum of thiosemicarbazone TSC-109.

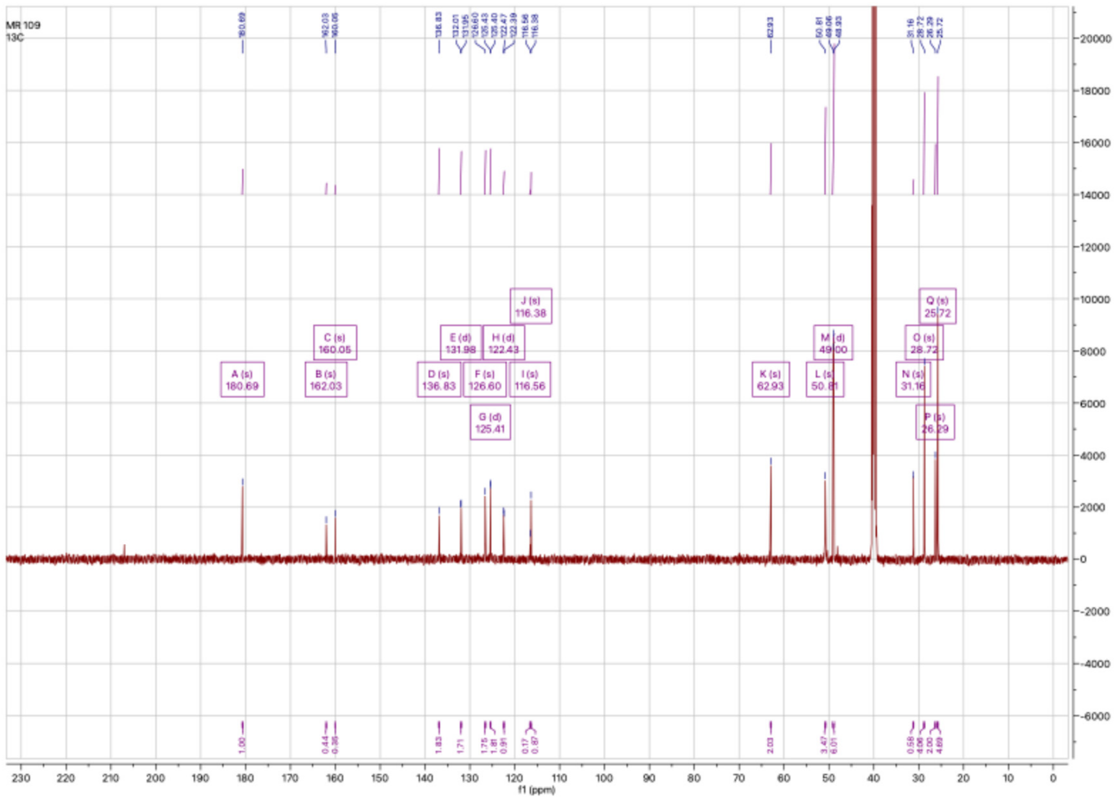

^{13}C-NMR spectrum of thiosemicarbazone TSC-109.

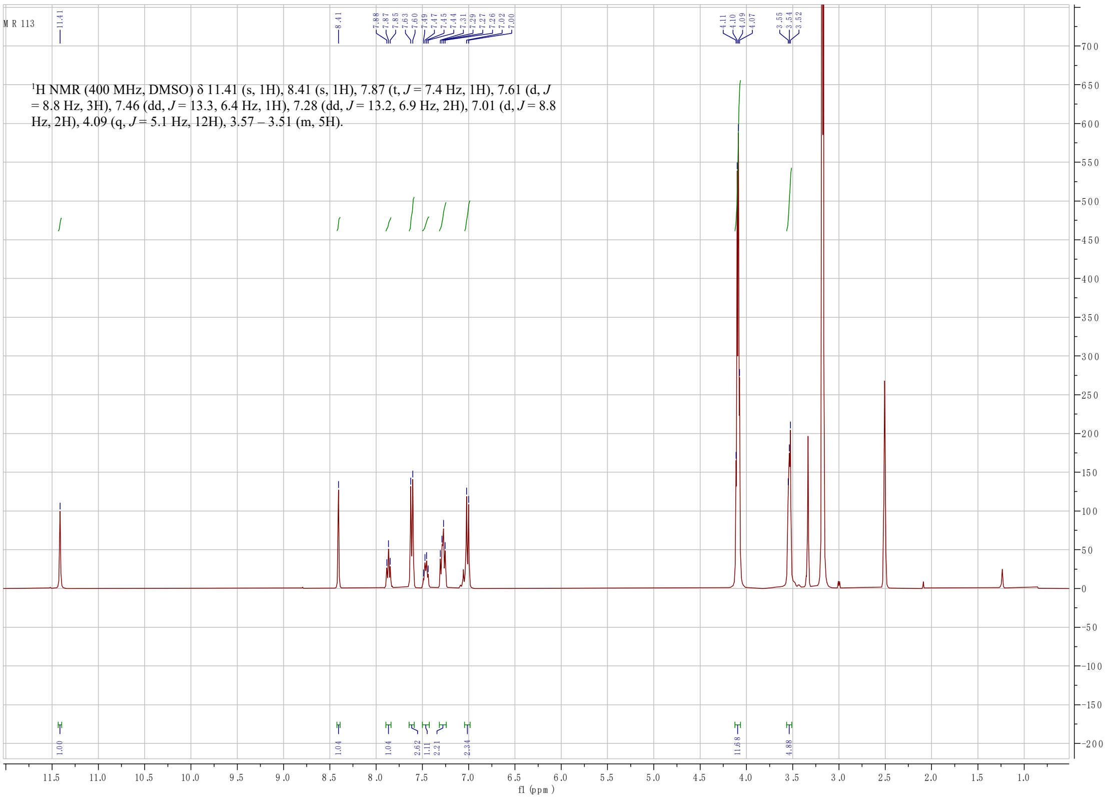

^{1}H-NMR spectrum of thiosemicarbazone TSC-113.

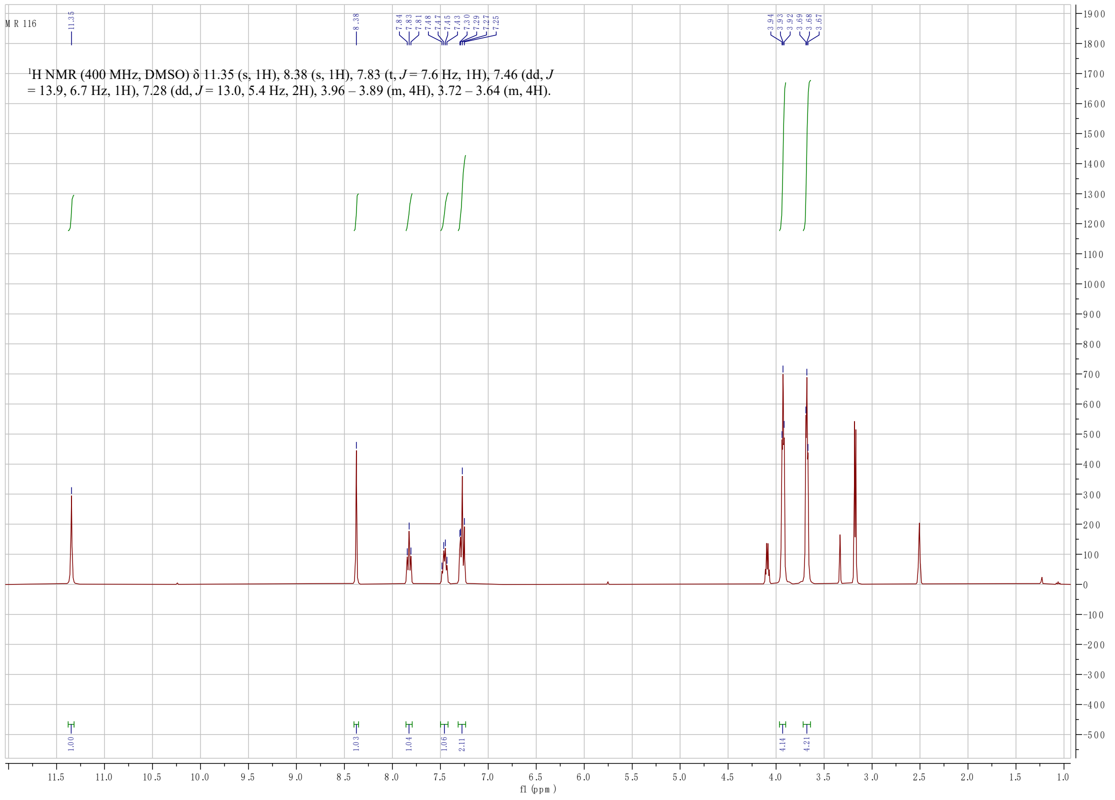

^{1}H-NMR spectrum of thiosemicarbazone TSC-116.

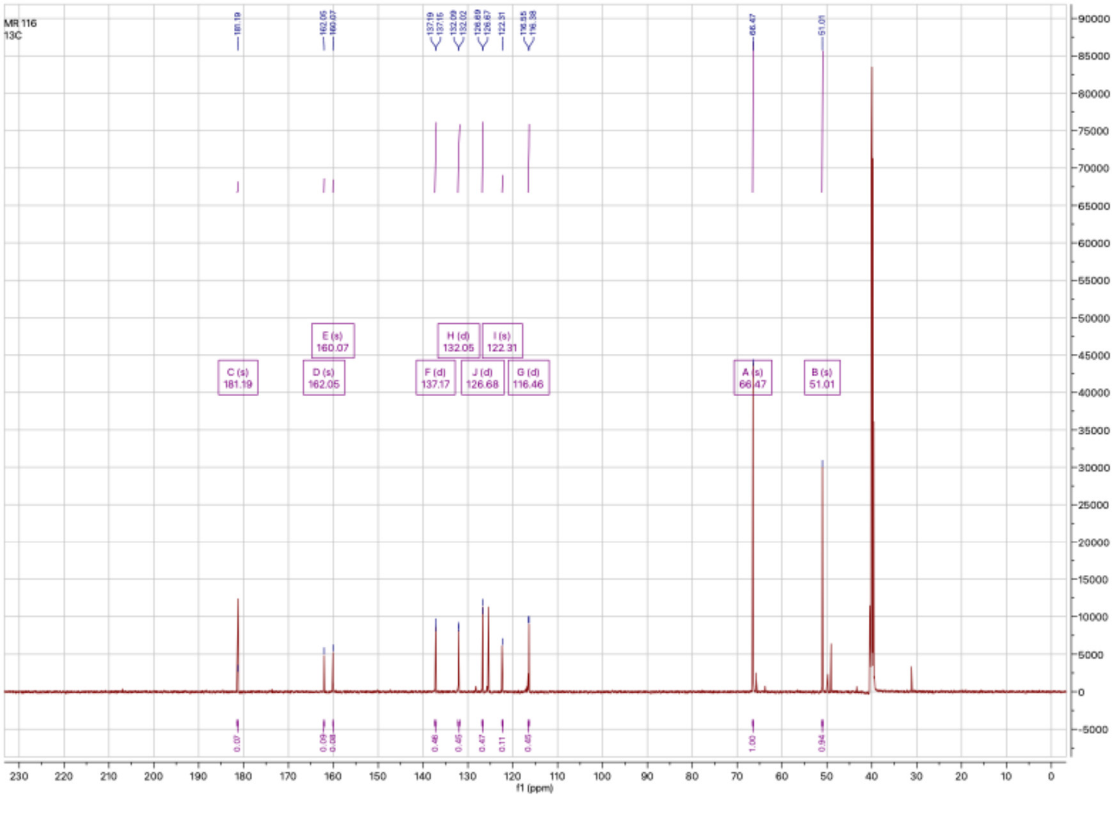

^{13}C-NMR spectrum of thiosemicarbazone TSC-116.

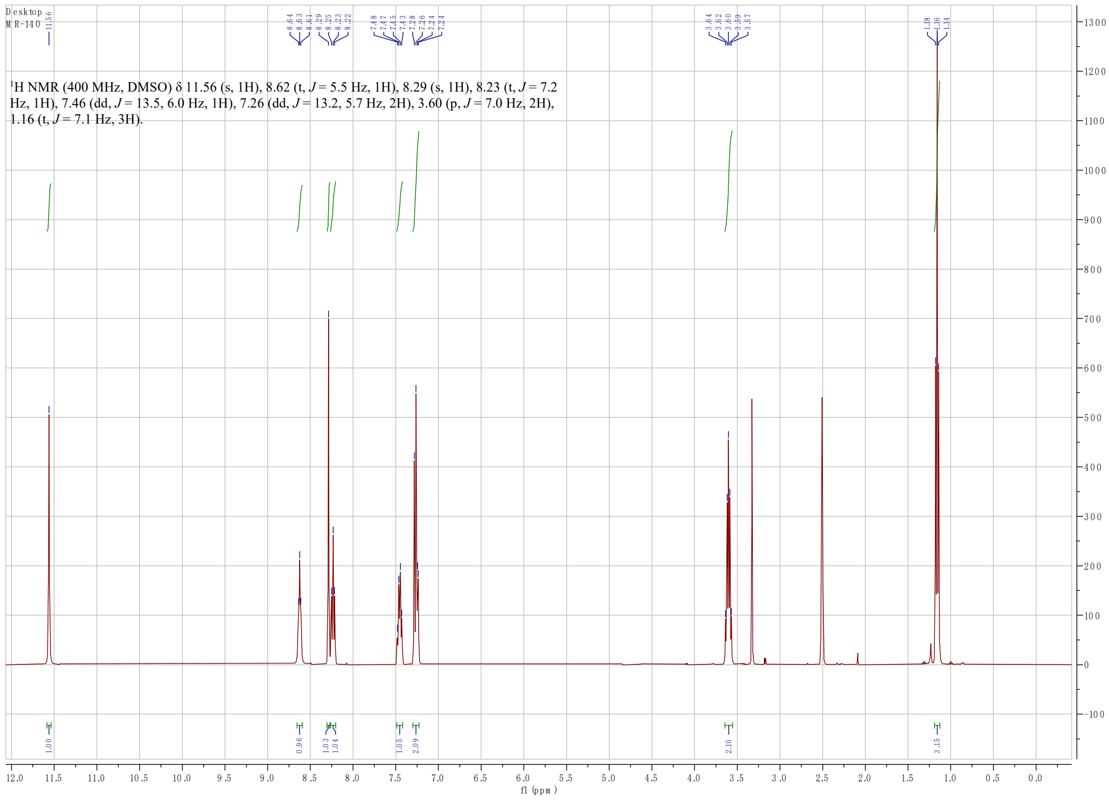

^{1}H-NMR spectrum of thiosemicarbazone TSC-140.

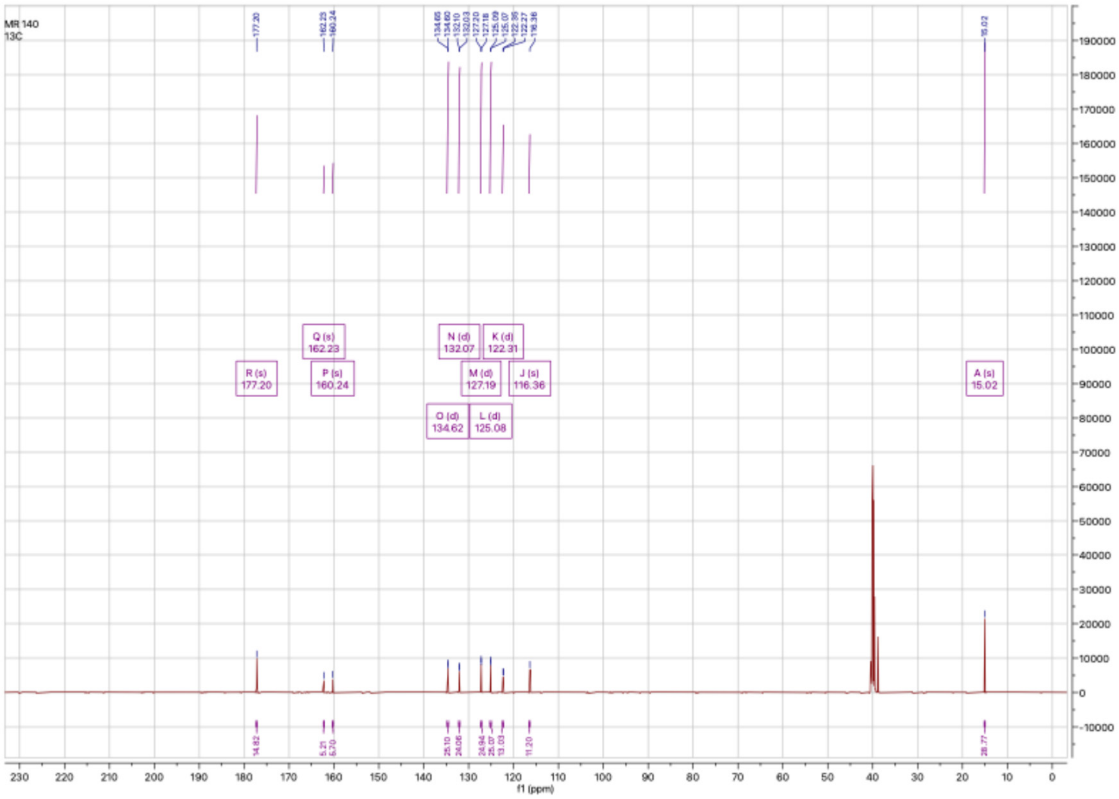

^{13}C-NMR spectrum of thiosemicarbazone TSC-140

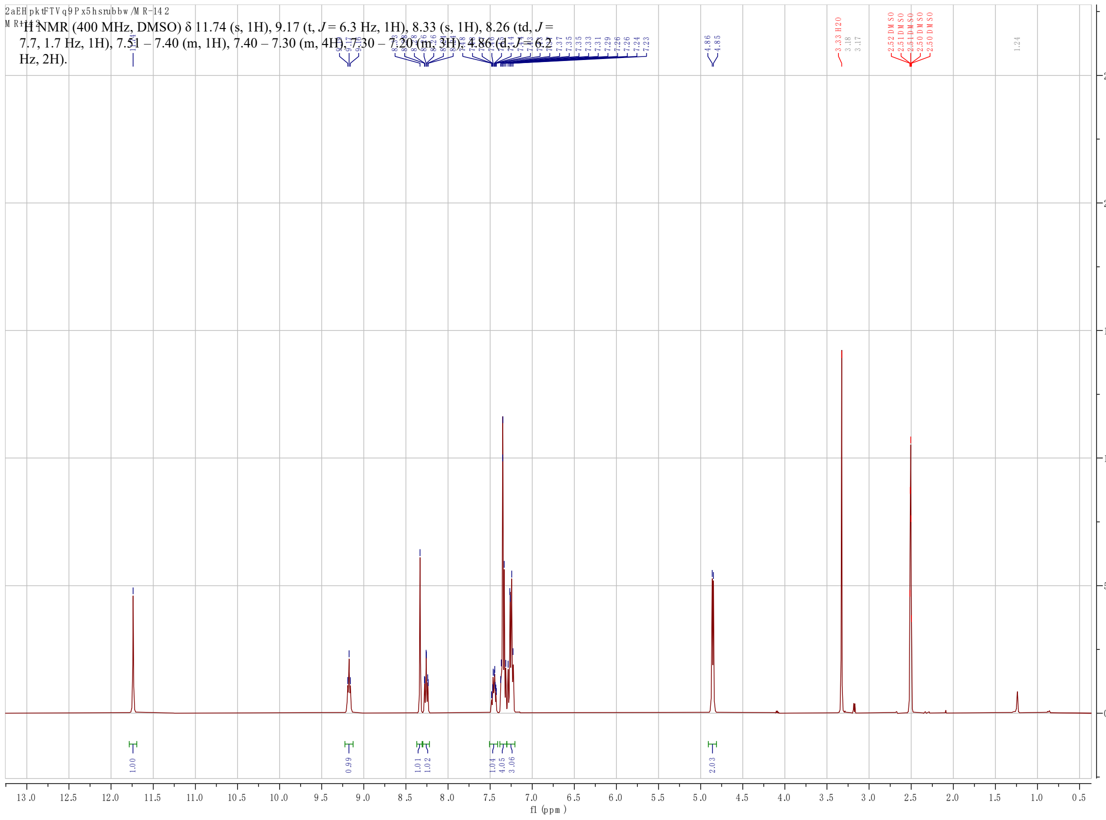

^{1}H-NMR spectrum of thiosemicarbazone TSC-142.

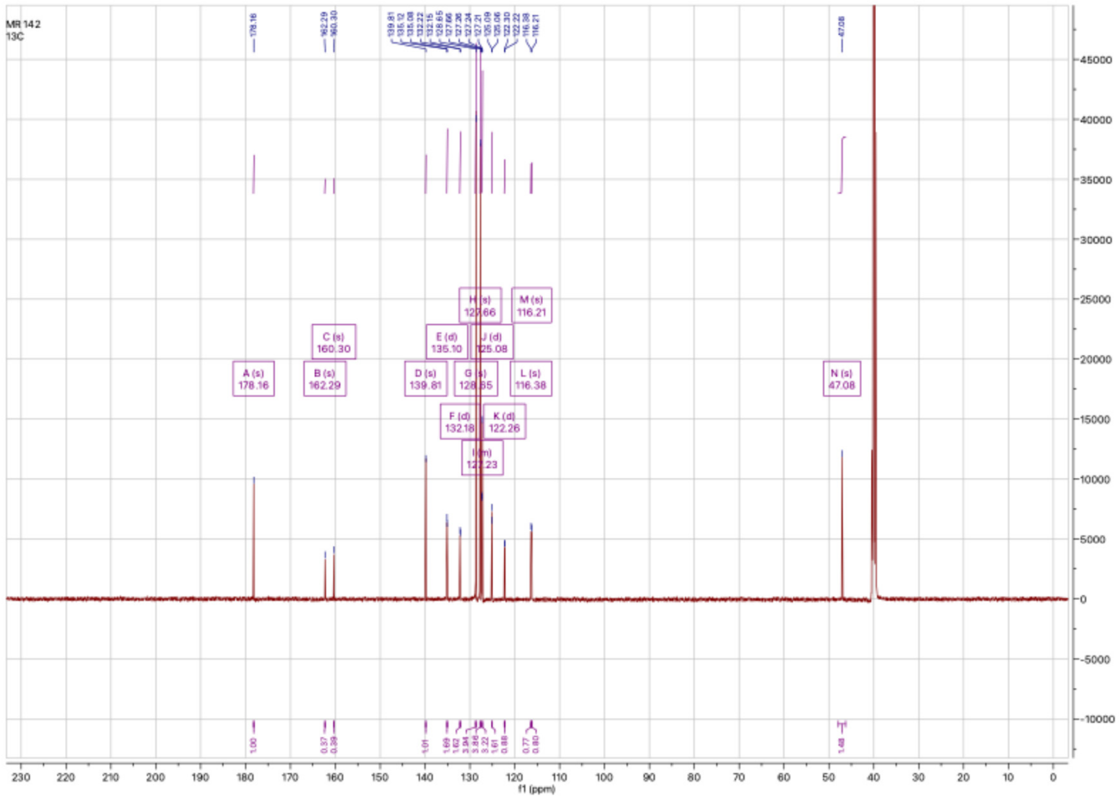

^{13}C-NMR spectrum of thiosemicarbazone TSC-142.

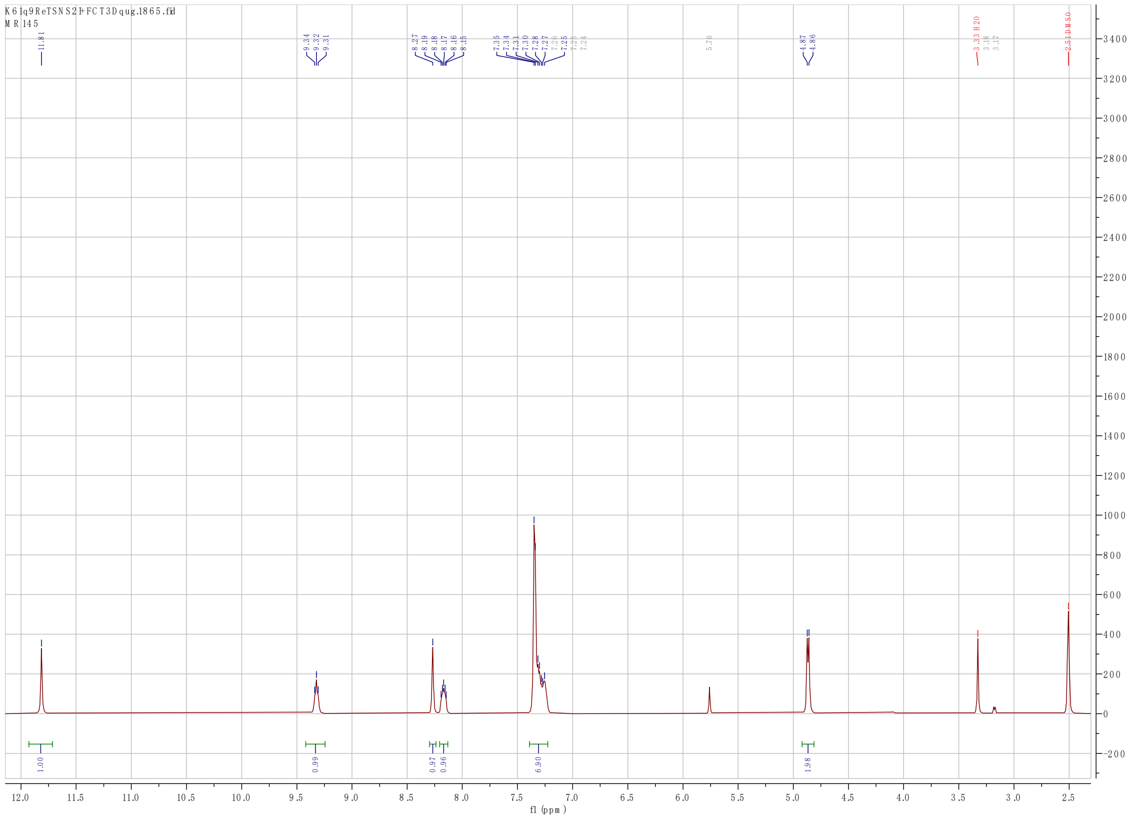

^{1}H-NMR spectrum of thiosemicarbazone TSC-145.

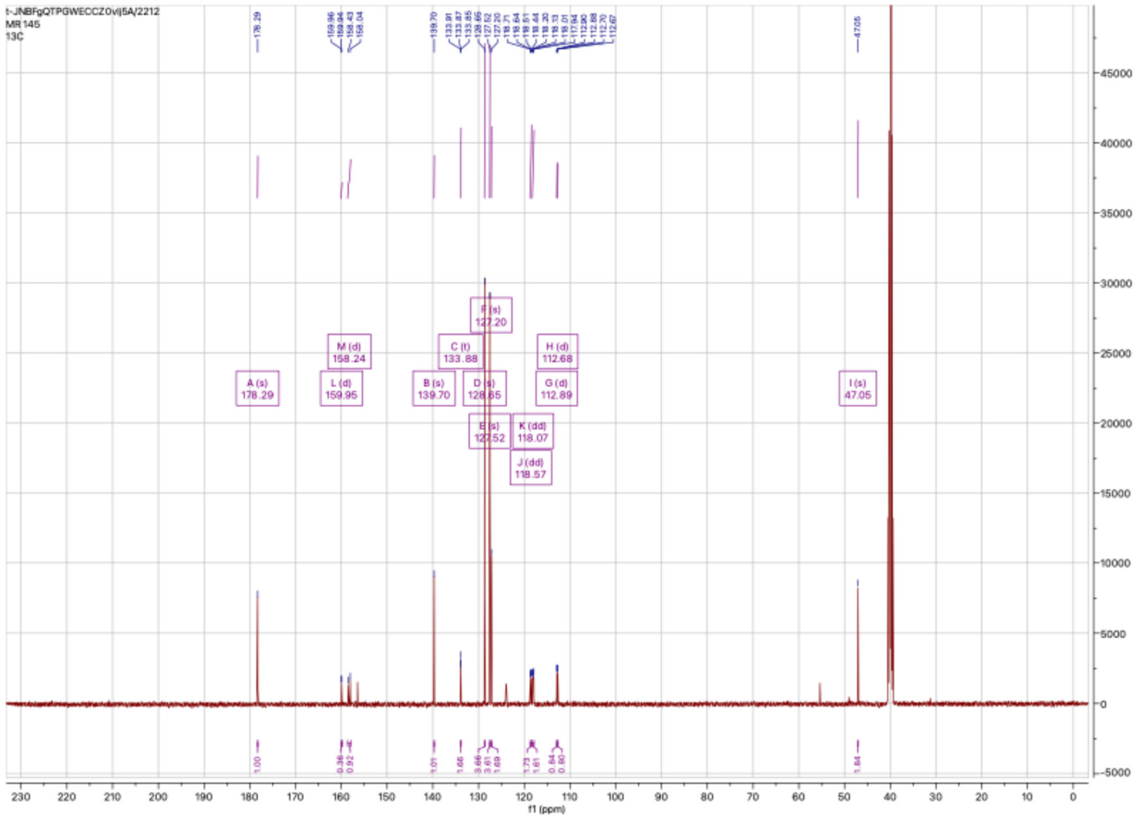

^{13}C-NMR spectrum of thiosemicarbazone TSC-145

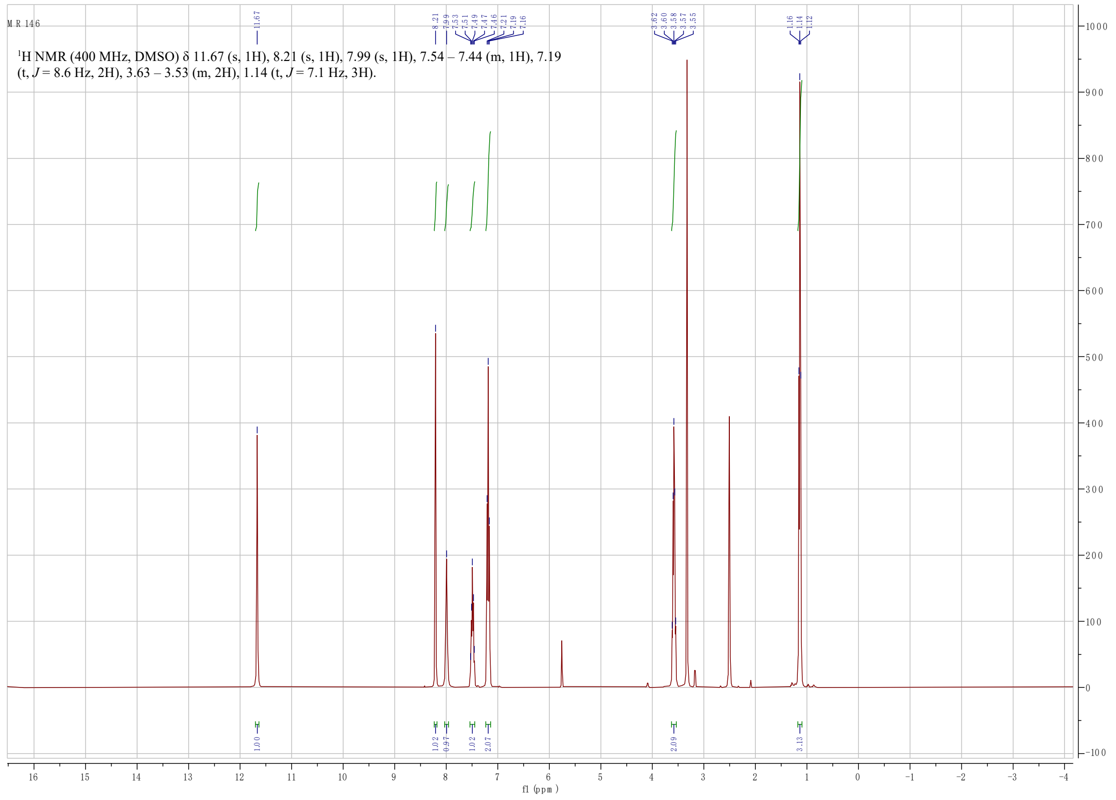

^{1}H-NMR spectrum of thiosemicarbazone TSC-146.

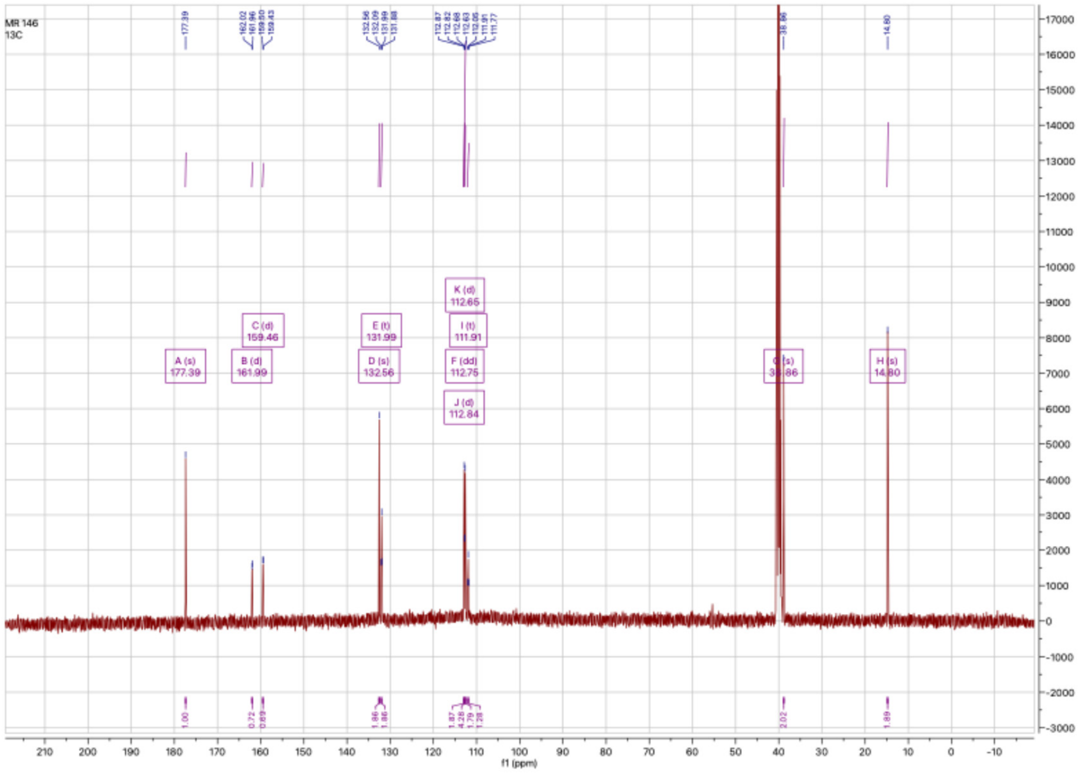

^{13}C-NMR spectrum of thiosemicarbazone TSC-146.

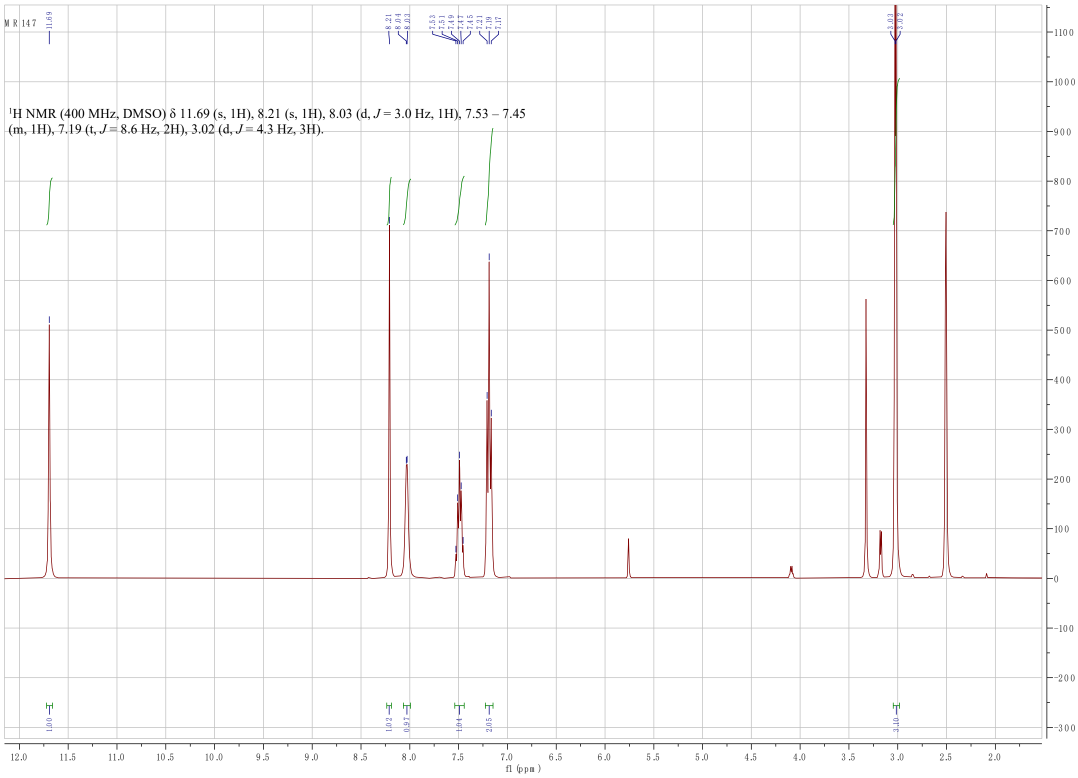

^{1}H-NMR spectrum of thiosemicarbazone TSC-147.

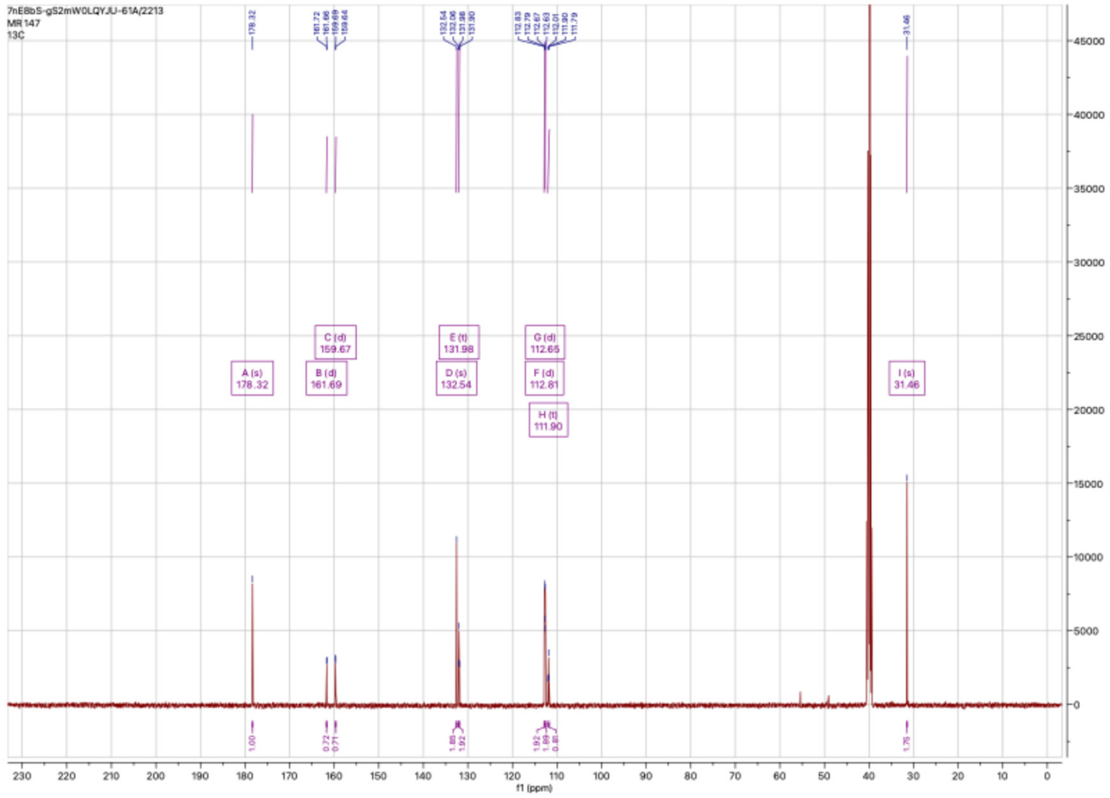

^{13}C-NMR spectrum of thiosemicarbazone TSC-147.

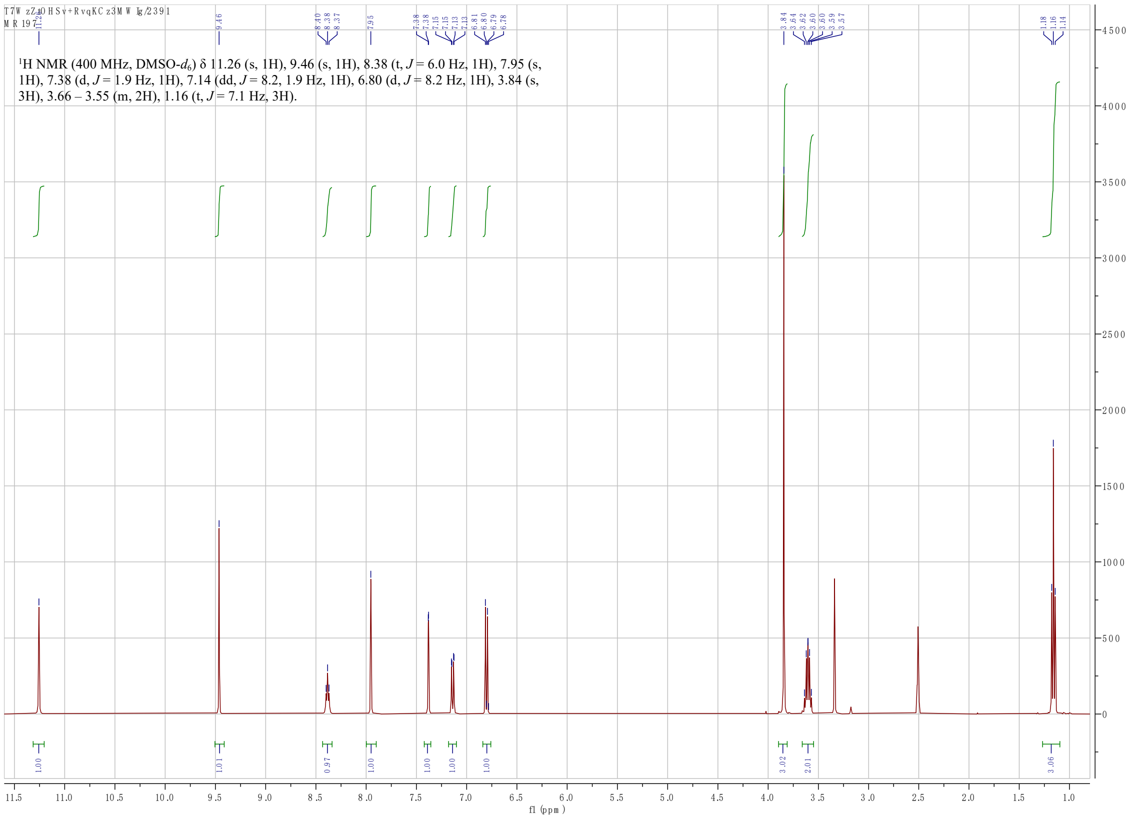

^{1}H-NMR spectrum of thiosemicarbazone TSC-197.

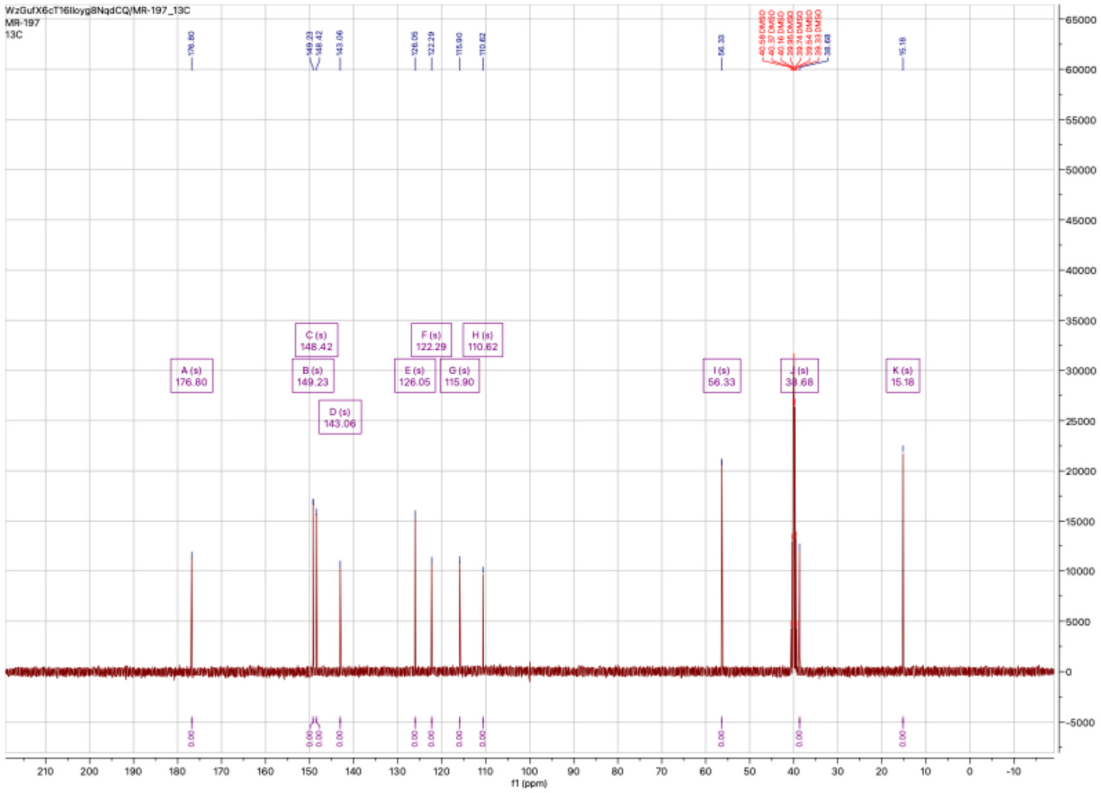

^{13}C-NMR spectrum of thiosemicarbazone TSC-197.

2.

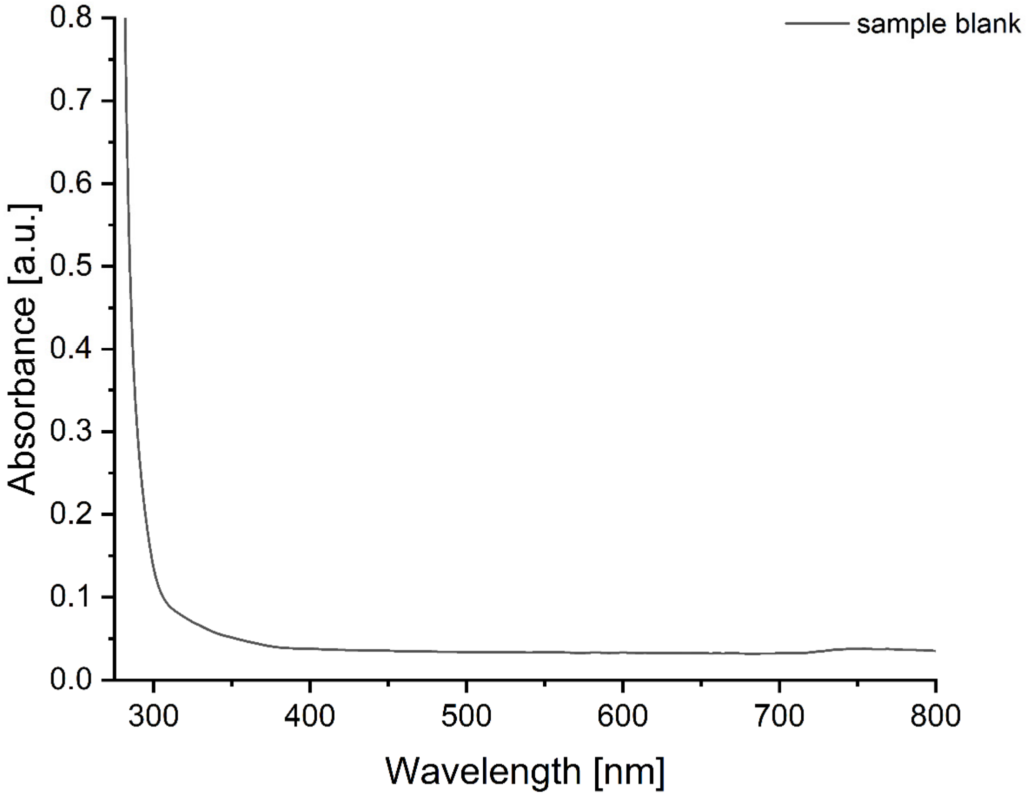

Absorption spectrum obtained for the solvent used in titration measurements (sample blank). Measurements were performed at room temperature.
